# The role of the LysR‐type transcription factor PacR in regulating nitrogen metabolism in *Anabaena* sp. PCC7120

**DOI:** 10.1111/ppl.70248

**Published:** 2025-05-05

**Authors:** Elisa Werner, Tuomas Huokko, Anita Santana‐Sánchez, Silvia Picossi, Lauri Nikkanen, Antonia Herrero, Yagut Allahverdiyeva

**Affiliations:** ^1^ Molecular Plant Biology, Department of Life Technologies University of Turku Turku Finland; ^2^ Instituto de Bioquímica Vegetal y Fotosíntesis, Consejo Superior de Investigaciones Científicas, Universidad de Sevilla Seville Spain

## Abstract

In the filamentous cyanobacterium *Anabaena* sp. PCC 7120, heterocyst formation is triggered by changes in the C/N‐ratio and relies on transcriptional reprogramming. The transcription factor PacR is considered a global regulator of carbon assimilation under photoautotrophic conditions, influencing the carbon concentrating mechanism and photosynthesis. It plays a role in balancing reducing power generation while protecting the photosynthetic apparatus from oxidative damage. However, PacR also binds to promoters of genes associated with heterocyst formation, although the underlying mechanisms remain unclear. To explore this, we studied the response of a PacR‐deletion mutant to a nitrogen source shift from ammonium to nitrate. The absence of PacR led to heterocyst formation in nitrate‐containing media, as well as reduced growth and chlorophyll content. We observed impaired nitrate uptake and disrupted ammonium assimilation via the GS/GOGAT‐cycle. This phenotype may stem from PacR‐mediated regulation of key genes of nitrogen and carbon metabolism as well as photosynthesis. An impact on photosynthesis is also apparent in the mutant, including a slight decrease in the size of the photo‐reducible Fed‐pool, suggesting that a shortage of reducing equivalents may contribute to nitrogen metabolism impairment.

## 1 INTRODUCTION

The filamentous cyanobacterium *Anabaena* sp. PCC7120 (hereafter *Anabaena*) is a model heterocyst‐forming strain that carries out oxygenic photosynthesis and CO_2_ fixation in vegetative (photosynthetic) cells, while performing N_2_ fixation in specialized cells called heterocysts. In the absence of combined nitrogen [e.g. ammonium (NH_4_^+^) or nitrate (NO_3_^−^)] about 10% of the vegetative cells differentiate into intercalary heterocysts within 24 h (Flores et al., 2019; Zeng & Zhang, 2022), which form at intervals of 10–15 vegetative cells along the filament (Flores et al., 2019; Nieves‐Morión et al., 2021). Heterocysts are specialized microoxic cells that fix atmospheric N_2_ into ammonia (NH_3_), a process catalyzed by the heterocyst‐specific O_2_‐sensitive nitrogenase enzyme (Flores et al., 2019; Nieves‐Morión et al., 2021). In the heterocyst, microoxic conditions are maintained by high respiration rates (Flores et al., 2019; Harish & Seth, 2020), the flavodiiron (Flv) 3B homo‐oligomer catalyzing O_2_ photoreduction (Ermakova et al., 2014), a non‐oxygen‐evolving Photosystem (PS) II with a reduced antenna size (Ferimazova et al., 2013; Harish & Seth, 2020; Magnuson, 2019) and the formation of a heterocyst envelope consisting of an inner glycolipid (Hgl) layer and an outer polysaccharide (Hep) layer (Cumino et al., 2007; Flores et al., 2019). Vegetative cells supply heterocysts with carbon in the form of sucrose, alanine and glutamate (Burnat et al., 2014). In heterocysts, the NH_4_^+^ produced during N_2_ fixation is incorporated into glutamate by glutamine synthetase (GS), producing glutamine, which is then transferred to the vegetative cell along with beta‐aspartyl‐arginine, the degradation product of cyanophycine (Flores et al., 2019). The process of heterocyst differentiation is highly complex and tightly regulated, with the primary factor being the C/N balance. In *Anabaena*, the primary trigger for differentiation is rising levels of 2‐oxoglutarate (2‐OG), which accumulates under a high C/N ratio and acts as a nitrogen starvation signal (Zeng & Zhang, 2022; Zhang et al., 2018). The global transcription factor NtcA binds 2‐OG and activates the expression of the master regulator of heterocyst formation, HetR, via the response regulator‐like factor NtcA (Flores et al., 2019; Zeng & Zhang, 2022; Zhang et al., 2018). NtcA and HetR together orchestrate the developmental process of heterocyst formation (Flores et al., 2019; Zeng & Zhang, 2022). Early in this process, PatS and PatX help to establish the initial heterocyst pattern (Flores et al., 2019). The DevH transcriptional factor regulates heterocyst‐specific genes at later stages of differentiation and plays a central role in regulating Hgl layer formation as well as expression of the *cox2* and *cox3* operons (functioning in O_2_ reduction) and the *nif* gene cluster (for N_2_‐fixation) (Zeng & Zhang, 2022). Other late‐stage regulators involved in heterocyst formation include the NtcA co‐activator PipX and HetN, which is crucial for pattern maintenance (Flores et al., 2019).

LysR‐type transcriptional regulators (LTTRs) are the largest known family of bacterial DNA‐binding proteins, playing a diverse role in regulating a wide range of biological processes. They may act as activators or repressors, often in response to a co‐inducer molecule (López‐Igual et al., 2012). Among the LTTRs subfamily, the CbbR factors specifically control genes involved in the Calvin‐Benson‐Bassham (CBB) cycle. In *Anabaena*, there are three CbbR‐like LTTRs. One of them, CmpR (*all0862*), activates the *cmp* operon (*alr2877‐alr2880*), which encodes an ABC transporter complex for HCO_3_^−^ transport. CmpR also controls its own expression in response to C_i‐_limitation and coordinates this with nitrogen availability through its response to NtcA (Picossi et al., 2015). Another member, CcmR (also known as NdhR), acts as a repressor for carbon transporters and its own expression (López‐Igual et al., 2012; Picossi et al., 2015). The third member, PacR (*rbcR, all3953*), is a global regulator. In *Anabaena*, PacR regulates a large number of genes associated with C_i_‐fixation [e.g. elements of the carbon concentrating mechanisms (CCM) and RuBisCo]. Remarkably, PacR was the first transcriptional factor described in cyanobacteria that can adjust the expression of photosynthetic genes (e.g. the core PSI reaction center *psaA* gene) to C_i_‐availability, with most genes showing upregulation by PacR under C_i_‐limitation (Picossi et al., 2015). Noteworthy, most photosynthetic genes targeted by PacR probably function in protection against reactive oxygen species, which can be generated due to C_i_‐limitation or exposure to high light. It has been proposed that PacR plays a crucial role in modulating the generation of reductive power based on C_i_‐availability while safeguarding the photosynthetic apparatus from oxidative stress. Notably, the PacR expression appears to be independent of the C/N regime (Picossi et al., 2015).

Despite PacR being identified as a global transcription factor in *Anabaena*, its role has primarily been studied in relation to the regulation of C_i_‐assimilation and photosynthetic genes, while its potential role in other parts of the global metabolic network, including nitrogen assimilation, remains largely unexplored. In this study, we analyzed a PacR deletion mutant using transcriptomics, combined with metabolic, physiological and photosynthetic measurements, during the shift from NH_4_^+^ to NO_3_^−^ as the available nitrogen source in the growth medium. Our results suggest that PacR plays a role in regulating nitrogen metabolism, including adaptation to NO_3_^−^ as a nitrogen source, thus influencing heterocyst differentiation in NO_3_^−^ medium. This study broadens our understanding of PacR's role in global regulatory processes, highlighting its contribution to adaptation to various nitrogen sources and involvement in heterocyst formation alongside other key transcriptional regulators.

## 2 MATERIALS AND METHODS

### 2.1 Strains and culture conditions

The ∆*pacR* deletion mutant of the *Anabaena* sp. PCC7120 wild type strain (CSS74) and control strain (CS) with a spectinomycin resistance cassette (CSS77) were described previously (Picossi et al., 2015). The starting cultures were maintained in BG11 medium buffered with 20 mM HEPES‐NaOH (pH 7.5) and supplemented with 25 ug/ml of spectinomycin. The cultures were grown at 30°C with 1% CO_2_, ~ 50 μmol photons m^−2^ s^−1^, and agitated at 80 rpm. Pre‐cultures were generated by harvesting cells from starting cultures after 4 days and resuspending cells at OD_750_ = 0.1. Pre‐cultures were then grown for 4 days in BG11_0_ medium buffered with 20 mM HEPES‐NaOH (pH 7.5) supplemented with 6 mM NH_4_Cl on day 0, and additional 3 mM NH_4_Cl were supplemented on days 2 and 3. Experimental cultures were generated similar to pre‐cultures and grown in BG11_0_ medium (pH 7.5) supplemented with either 3 mM NH_4_^+^ (on day 1, 2 and 3 for growth experiments) or 17.6 mM NO_3_^−^ as the nitrogen source.

### 2.2 Chl *a* determination and heterocyst frequency

Chl *a* was extracted from cells with 90% methanol and the extinction coefficient factor 12.7 (Meeks & Castenholz, 1971). For counting of heterocysts, Alcian Blue was used to stain the polysaccharide layer of the heterocyst envelope as previously described (Santana‐Sánchez et al., 2023). Stained samples were visualized using a Wetzlar light microscope (Leitz) and x 400 magnification micrographs were taken via a mounted camera (Leica). Images were processed using the Leica Application Suite (Leica Microsystems). For each sample, 1000–2000 cells were counted.

### 2.3 Photosynthetic activity measurements

#### 2.3.1 Chl fluorescence and absorbance

A pulse amplitude modulated fluorometer Dual‐PAM‐100 (Walz) was used to simultaneously monitor Chl *a* fluorescence and P700 absorbance. Cells were harvested and resuspended in fresh BG11 medium to a Chl *a* concentration of 15 μg mL^−1^ and acclimated 1 h under growth conditions. Prior to measurements, the samples were dark‐adapted for 10 min. The effective yield of PSII [Y(II)] and PSI [Y(I)], as well as the acceptor side limitation of PSI [Y(NA)] and donor side limitation [Y(ND)], were determined as described previously (Huokko et al., 2017). Actinic light (630 nm, 50 μmol photons m^−2^ s^−1^), saturating pulses (300 ms, 5 000 μmol photons m^−2^ s^−1^), and strong far‐red light (720 nm, 75 W m^−2^) were applied during analysis.

#### 2.3.2 Determination of ferredoxin (Fed) redox changes

A Dual‐KLAS‐NIR spectrophotometer (Walz) was used to measure the absorbance differences at 780–820, 820–870, 840–965 and 870–965 nm. Cells were harvested and resuspended in fresh BG11 medium to a Chl *a* concentration of 20 μg mL^−1^ and acclimated 1 h under growth conditions. Prior to measurements, the samples were dark‐adapted for 10 min. Absorbance differences of the four wavelength pairs were measured during 3 s of red actinic illumination (3000 μmol photons m^−2^ s^−1^), with a multiple turnover (MT) pulse applied after 200 ms to fully reduce the Fed‐pool. This was followed by a 4 s dark period and 10 s of far‐red illumination (intensity setting 20), with a MT pulse administered at the end of the illumination period to fully oxidize P700. Measurements using the NIRMAX script (Klughammer & Schreiber, 2016; Schreiber, 2017) were conducted, and the P700 and Fed signals deconvoluted using model spectra measured for *Anabaena* as previously described (Santana‐Sánchez et al., 2023). The redox‐changes were then normalized according to the maximal redox‐changes determined with the NIRMAX script. The size of the photo‐reducible Fed pool was calculated from about 100 datapoints of the smoothed and baseline‐corrected data at the maximal reduction phase during actinic light illumination using the NIRMAX script.

#### 2.3.3 Membrane Inlet Mass Spectrometry

*In vivo* measurements of ^16^O_2_ (m z^−1^ = 32), ^18^O_2_ (m/z = 36) and CO_2_ (m/z = 44) fluxes were monitored using Membrane Inlet Mass Spectrometry (MIMS) as described previously (Santana‐Sánchez et al., 2023). Cells were harvested after 48 h and resuspended in fresh BG11 medium, adjusted to Chl *a* 10 μg mL^−1^ and acclimated for 1 h under growth conditions prior to measurements. For all MIMS measurements, gas exchange was monitored for 4 min in darkness, followed by 5 min of high light illumination (500 μmol photons m^−2^ s^−1^) and for an additional 2 min in the dark.

### 2.4 Determination of nitrogen uptake and intracellular carbon and nitrogen content

NO_3_^−^ concentration in the growth media was determined spectrophotometrically (Rice et al., 2012). To determine intracellular C/N ratio, cells were harvested 48 h after the shift to NO_3_^−^ as nitrogen source and 10 mg of biomass was lyophilised. Total carbon and nitrogen analysis was conducted using a FLASH 2000 Organic Elemental Analyzer (Thermo Scientific).

### 2.5 Metabolite extraction and quantification

Cells were harvested by filtration on Millipore HATF nitrocellulose membranes (0.45 μm) from cultures grown for 4 days with NH_4_^+^ as the nitrogen source, as well as at 1 h and 12 h after shifting to NO_3_^−^ containing growth medium. Filters were first frozen in liquid N_2_, then harvested cells were resuspended in PBS buffer, pelleted and stored at −80°C. Metabolites were extracted by adding 400 μL of cold extraction solvent (acetonitrile:methanol:H_2_O; 40:40:20) to each sample, followed by three cycles of sonication (60 sec, Elma Elmasonic P) and vortexing (120 sec). The samples were then centrifuged at 15339 x *g* for 20 min at +4°C, after which the supernatants (350 μL) were transferred to evaporation tubes and dried under a N_2_‐stream. Dried samples were resuspended in 50 μL cold extraction solvent and transferred to HPLC autosampler glass vials. The analysis was performed by injecting 2 μL of sample extract into a Thermo Vanquish UHPLC coupled with a Q‐Exactive Orbitrap quadrupole mass spectrometer equipped with a heated electrospray ionization (H‐ESI) source probe (Thermo Fischer Scientific). Chromatographic separation of metabolites was done using a SeQuant ZIC‐pHILIC (2.1 × 100 mm, 5 μm particle) column (Merck), which was maintained at +40°C. Scanning was performed in mass range 55 to 825 m/z. Gradient elution was carried out with acetonitrile and 20 mM ammonium hydrogen carbonate in water, adjusted to pH 9.4, as mobile phases. TraceFinder 4.1 software (Thermo Fischer Scientific) was used for data analysis.

### 2.6 RNA isolation, sequencing and enrichment analysis

Cells were harvested by filtration on Millipore HATF nitrocellulose membranes (0.45 μm) from cultures grown for 4 days with NH_4_^+^ as the nitrogen source, as well as at 1 h and 12 h after shifting to NO_3_^−^ containing growth medium. Cells were resuspended in RNA‐resuspension buffer (Walter et al., 2016), centrifuged for 3 min at 4°C and then the pellet was then frozen in liquid N_2_. Total RNA was isolated using the hot‐phenol method (Walter et al., 2016), with the incubation step in phenol:chloroform:iso‐amyl alcohol performed at 65°C instead of 95°C. Finally, the samples were resuspended in 25 μL of Milli‐Q Water. DNAse treatment was performed with the Invitrogen kit. RNA concentration was measured with DS‐11+ spectrophotometer (DeNovix) and 3 ug of RNA from each sample was sent to BGI (China) for RNA sequencing. RNA quality was checked using agarose gel electrophoresis, followed by rRNA depletion, yielding acceptable RIN values (7.2–8.2). Three biological replicates were measured for each time point and strain. Experimental quality control evaluated via PCA (Figure S9) confirmed the data's reliability.

The RNA was sequenced using DNBseq with a read length of PE 100 bp and data was analyzed with the Chipster software. Initial trimming of the reads was performed using FastX, followed by alignment with the genome of *Anabaena* (downloaded from EnsemblBacteria (bacteria.ensembl.org), nostoc_sp_pcc_7120_fachb_418_gca_000009705, version ASM970v1_, current ENSEMBL release, October 2023) using TopHat2. Aligned reads per gene were then counted with HTSeq. Differential expression analysis was conducted using DESeq2 (log_2_FC ≧ I1I, *p* < 0.05). Enrichment analysis was performed using the R‐package clusterProfiler.

## 3 RESULTS

### 3.1 Phenotype of the Δ*pacR* mutant under different nitrogen sources

**FIGURE 1:**
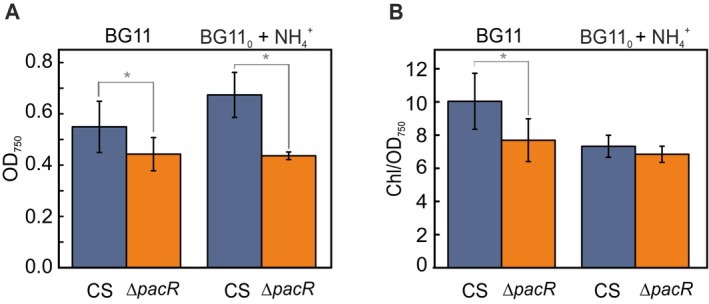
OD and Chl/OD ratio of the ∆*pacR* mutant 48 h after the shift to NO_3_^−^ or 3 mM NH_4_^+^ (day 0 and day 1) as the sole nitrogen source. **A**. OD_750_; **B**. Chl/OD ratio. Values are means ± SD; *n =* 3–14 biologically independent experiments; statistically significant differences (*p* < 0.05) compared to the CS are indicated by an asterisk. Statistical analysis was performed using a two‐tailed Type 2 t‐test.

The growth of the ∆*pacR* mutant and the CS was compared under various nitrogen sources. The ∆*pacR* mutant demonstrated significantly slower growth when either NO_3_^−^ (Figure S1A) or NH_4_^+^ was used as the nitrogen source (Figure S1B). At 48 h in NO_3_^−^‐containing medium, the ∆*pacR* mutant reached only 80.4 ± 16.5% of the OD_750_ of the CS (Figures 1A and S1A). Similarly, when grown with NH_4_^+^, the mutant achieved only 72.3 ± 11.2% of the OD_750_ of the CS at 48 h (Figures 1A and S1B). To further assess cellular composition, we examined the Chl/OD_750_ ratio. In NO_3_^−^ containing medium, the ∆*pacR* mutant exhibited a 21.6% reduction in the Chl/OD_750_ ratio compared to the CS at 48 h (Figures 1B and S2A). However, this ratio was not significantly different between strains grown in NH_4_^+^‐ containing medium (Figures 1B and S2B).

**FIGURE 2:**
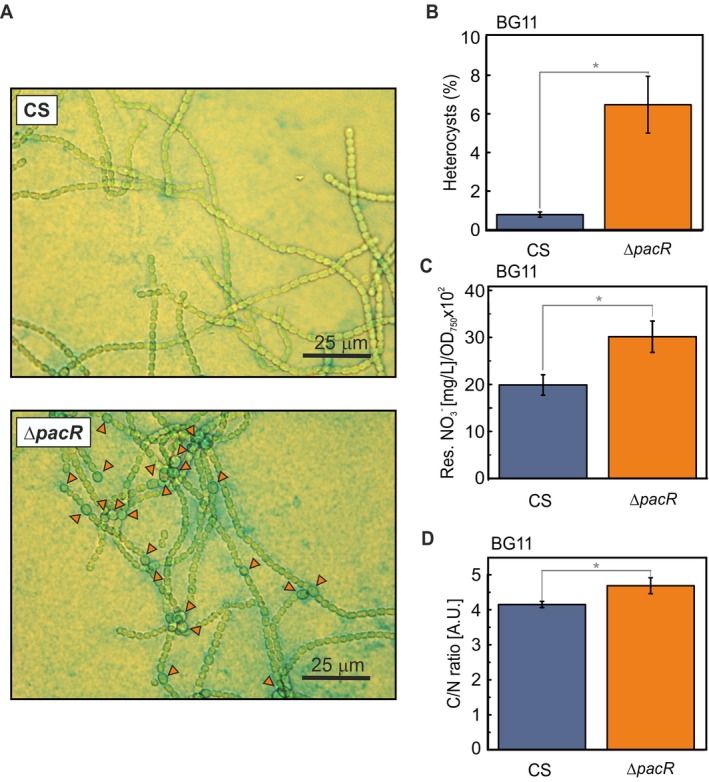
Heterocyst formation, NO_3_^−^uptake and C/N balance of the ∆*pacR* mutant 48 h after the shift to NO_3_^−^. **A**. Representative light‐microscopy images; **B**. Number of heterocysts. 1000–2000 cells were counted for each biological replicate; **C**. Residual NO_3_^−^ in the growth medium; **D**. C/N ratio. Values are means ± SD; *n =* 3–14 biologically independent experiments; statistically significant differences (p < 0.05) compared to the CS are indicated by an asterisk. Statistical analysis was performed using a two‐tailed Type 2 t‐test.

Interestingly, the ∆*pacR* mutant showed a significantly higher proportion of heterocysts (7.8 ± 1.4%, *p* < 0.05) compared to the CS (0.6 ± 0.5%) when cultured in NO_3_^−^‐containing medium for 48 h (Figure 2A, B; Table S1). In contrast, neither strain exhibited substantial heterocyst differentiation in NH_4_^+^‐containing medium, with heterocysts frequencies of 0.05 ± 0.04% (CS) and 0.26 ± 0.36% (∆*pacR*) (Table S1). These findings suggest that PacR is required for the suppression of heterocyst differentiation in the presence of NO_3_^−^.

Next, we examined the ∆*pacR* mutant during the transition from NH_4_^+^ to NO_3_^−^ as the nitrogen source to investigate the mechanisms underlying its increased heterocyst differentiation. We focused on time points up to 48 h after transition, as this is when heterocyst formation becomes established (Flores et al., 2019; Zeng & Zhang, 2022) and key phenotypic differences, like delayed growth and altered Chl content became evident. During the transition, the Δ*pacR* mutant demonstrated impaired NO_3_^−^‐uptake, showing 1.5 ± 0.2 times more residual NO_3_^−^/OD_750_ in the growth medium compared to the CS at 48 h (Figure 2C). This phenotype was maintained for the total culturing time of 120 h (Figure S3). Importantly, organic elemental analysis showed that the ∆*pacR* mutant had an increased C/N ratio, reflecting a 14.3 ± 4.9% reduction in total nitrogen content compared to the CS (Figure 2D; Table S2), suggesting that the ∆*pacR* mutant experiences nitrogen deprivation.

### 3.2 Photosynthetic characterization of the ∆ *pacR* mutant

**FIGURE 3:**
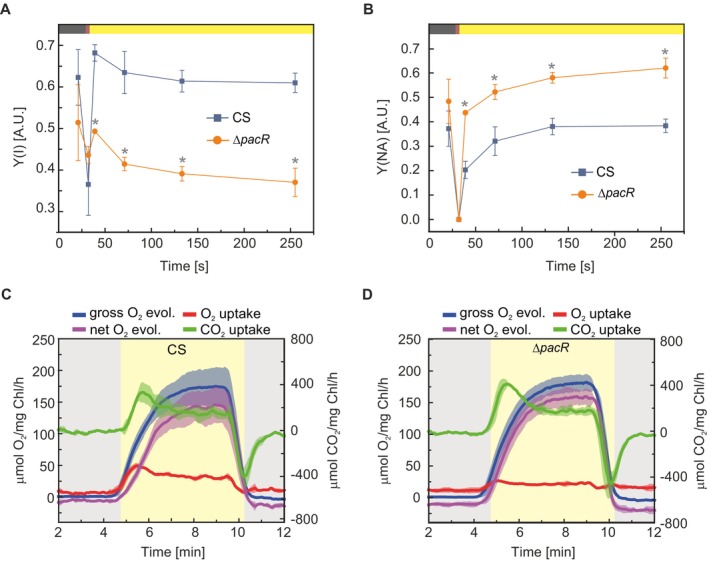
Photosynthetic performance of the *∆pacR* mutant and the CS 48 h after the shift to NO_3_^−^. **A**. The effective PSI yield [Y(I)]; **B**. The acceptor side limitation of PSI [Y(NA)]. A and B were measured in darkness (black bars), under illumination with far‐red light (red bars) and with 50 μmol photons m^−2^ s^−1^ of actinic white light (yellow bars); **C**. and **D**. O_2_ and CO_2_ exchange rates of the CS (C) and the ∆*pacR*‐mutant (D). Gas exchange rates were recorded in darkness (grey bars) and under illumination with 500 μmol photons m^−2^ s^−1^ of actinic white light (yellow bars). Values are means ± SD; *n =* 3 biologically independent experiments; statistically significant differences (p < 0.05) compared to the CS are indicated by an asterisk. Statistical analysis was performed using a two‐tailed Type 2 t‐test.

Next, we examined the photosynthetic capacity of the ∆*pacR* mutant cultured in NO_3_^−^ for 48 h. The effective yield of PSI, Y(I), was reduced in the mutant compared to the CS (Figure 3A) and acceptor‐side limitation of PSI, Y(NA), was increased (Figure 3B). This was in line with a pronounced reduction in PSI re‐oxidation, as detected with DKN measurements (Figure S5C).

Although the effective PSII yield, Y(II), was higher in the mutant, this difference was not statistically significant (Figure S4A). Additionally, donor side limitation Y(ND) of PSI remained unaffected (Figure S4B). The measurements with DKN demonstrated a slight but significant decrease in the pool size of photo‐reducible Fed and no changes in redox kinetics of Fed in the mutant (Figure S5).

For a more detailed assessment of the activity of the photosynthetic apparatus, we monitored real‐time gas exchange fluxes via Membrane Inlet Mass Spectrometry (MIMS) and used the ^18^O_2_ isotope to differentiate oxygen uptake from evolution. The illumination of the CS led to rapid O_2_‐photoreduction, primarily attributed to Flvs (Santana‐Sánchez et al., 2023), reaching a maximum of 40.9 ± 3.9 μmol mg^−1^ Chl *a* h^−1^ (*p* < 0.05) and plateaued at 23.1 ± 2.7 μmol mg^−1^ Chl *a* h^−1^ (p < 0.05). However, the ∆*pacR* mutant exhibited significantly lower O_2_‐photoreduction, reaching a maximum of 13.0 ± 3.0 μmol mg^−1^ Chl *a* h‐1, with an average plateau level of 7.3 ± 5.0 μmol mg^−1^ Chl *a* h^−1^ (p < 0.05), indicating impaired light‐induced O_2_‐uptake in the ∆*pacR* mutant. This is in line with the higher net O_2_ evolution observed in the mutant compared to the CS, calculated as the difference between gross O_2_ production and O_2_ photoreduction, with values of 142.1 ± 26.1 and 159.4 ± 11.1 μmol mg^−1^ Chl *a* h^−1^ for the CS and ∆*pacR*, respectively. Gross photosynthetic O_2_ evolution exhibited no significant difference between the CS and ∆*pacR*. Additionally, dark respiration as well as maximal and steady‐state CO_2_ uptake were somewhat higher in the mutant (Figure 3C, D; Table S3).

### 3.3 Metabolic analysis of the ∆ *pacR* mutant

**FIGURE 4:**
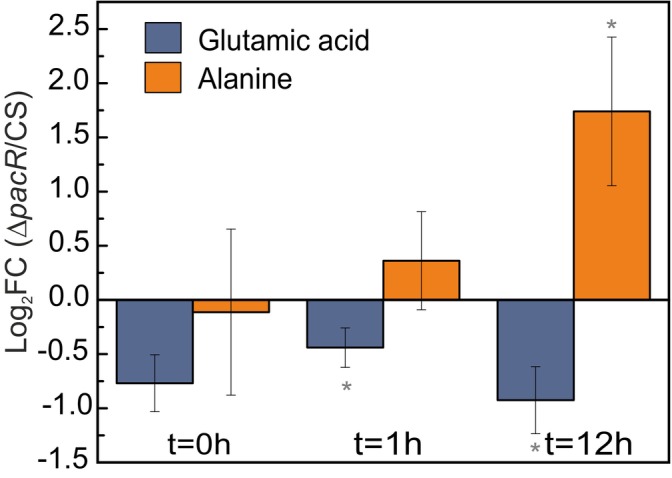
Metabolic analysis of glutamic acid and alanine in the *∆pacR* mutant relative to the CS at 0 h and 1 h or 12 h after the shift to NO_3_^−^. Values are mean ratios of ion counts shown as log_2_FC ± SD; *n =* 3–6 biologically independent experiments; statistically significant differences (p < 0.05) compared to the CS were calculated based on the normalized total ion count and are indicated by an asterisk. Statistical analysis was performed using a two‐tailed Type 2 t‐test.

To understand the metabolic responses associated with heterocyst formation in the ∆*pacR* mutant cultured with NO_3_^−^, we monitored the levels of several key metabolites from the tricarboxylic acid (TCA)‐, glutamine synthase / Fed‐dependent glutamate synthase (GS/GOGAT)‐ and ornithine‐ammonia (OAC)‐ cycles at three time points: in the presence of NH_4_^+^ as the only supplemented nitrogen source in the medium just before the shift (t = 0 h), and 1 h and 12 h after the shift to NO_3_^−^‐containing medium. Changes in metabolite levels in the ∆*pacR* mutant in relation to the CS are shown for each time point (Figures 4 and S6), as well as changes of metabolite levels in relation to t = 0 h for each strain (Figure S7).

The differences in metabolite levels between these two strains become more pronounced with longer exposure to NO_3_^−^. Most metabolites showed lower levels in ∆*pacR* compared to the CS, except for alanine, which increased significantly in the ∆*pacR* mutant at t = 12 h (Figures 4 and S6). Strikingly, glutamate levels were lower in ∆*pacR* across all time points, with statistical significance at t = 1 h and t = 12 h. Glutamine showed a similar trend but without significant changes (Figure S6). Upon exposure to NO_3_^−^, 2‐OG levels increased in both strains compared to t = 0 h, which is expected during N step‐down. However, this rise was statistically significant only in the CS, while no significant differences were observed between the strains or in the 2‐OG/Glu ratios in ∆pacR at any time point Figures S6, S7, and S8).

While not statistically significant, the Δ*pacR* mutant exhibited lower levels of aspartate, arginine and ornithine (intermediates of the OAC and cyanophycine synthesis) across all time points, whereas asparagine levels were higher (Figure S6). Pyruvate content peaked 1 h after the shift in both strains (Figure S7), but remained lower in ∆*pacR* compared to the CS. Similarly, citric acid, fumarate and malate (intermediates of the TCA cycle) were less abundant in ∆*pacR* than in the CS (Figure S6).

### 3.4 RNA‐seq analysis of the ∆ *pacR* mutant

To elucidate the differential expression of genes due to the deletion of *pacR*, we performed RNA‐seq analysis of the CS and the Δ*pacR* mutant grown in the presence of NH_4_^+^ as the only supplemented nitrogen source in the medium and sampled just before the shift (t = 0 h) as well as 1 h and 12 h after the shift to medium supplemented with NO_3_^−^ as the only nitrogen source (Appendix [Link], [Link], [Link]). At t = 0 h, 705 genes were found to be upregulated and 774 genes downregulated, at t = 1 h, 639 genes were upregulated and 707 genes downregulated and at t = 12 h, 1344 genes were upregulated and 1034 genes downregulated in the Δ*pacR* mutant in relation to the CS (Appendix S1). Thus, up to ~38% of the gene transcripts of *Anabaena* (RefSeq: GCF_000009705.1) were differentially expressed in the Δ*pacR* mutant at any given time. Differentially expressed genes were found *e*.*g*. in the categories of global transcriptional regulators, nitrogen uptake and heterocyst formation, the GS/GOGAT‐cycle, the OAC and cyanophycin synthesis, carbon uptake as well as the photosynthetic machinery. An enrichment analysis of differentially expressed genes showed significant enrichment of nitrogen transporters 1 h after the shift, followed by an enrichment of photosynthetic antenna proteins at 12 h (Figure S10; Appendix S3). The genes discussed below were selected based on their known function and relevance in the context of other data. Particular emphasis was placed on nitrogen metabolism, as well as photosynthesis and carbon fixation. Genes are categorized as either slightly (log_2_FC = 1–2), intermediately (log_2_FC = 2–4) or highly (log_2_FC >4) differentially expressed.

#### 3.4.1 Transcriptional regulators

**FIGURE 5:**
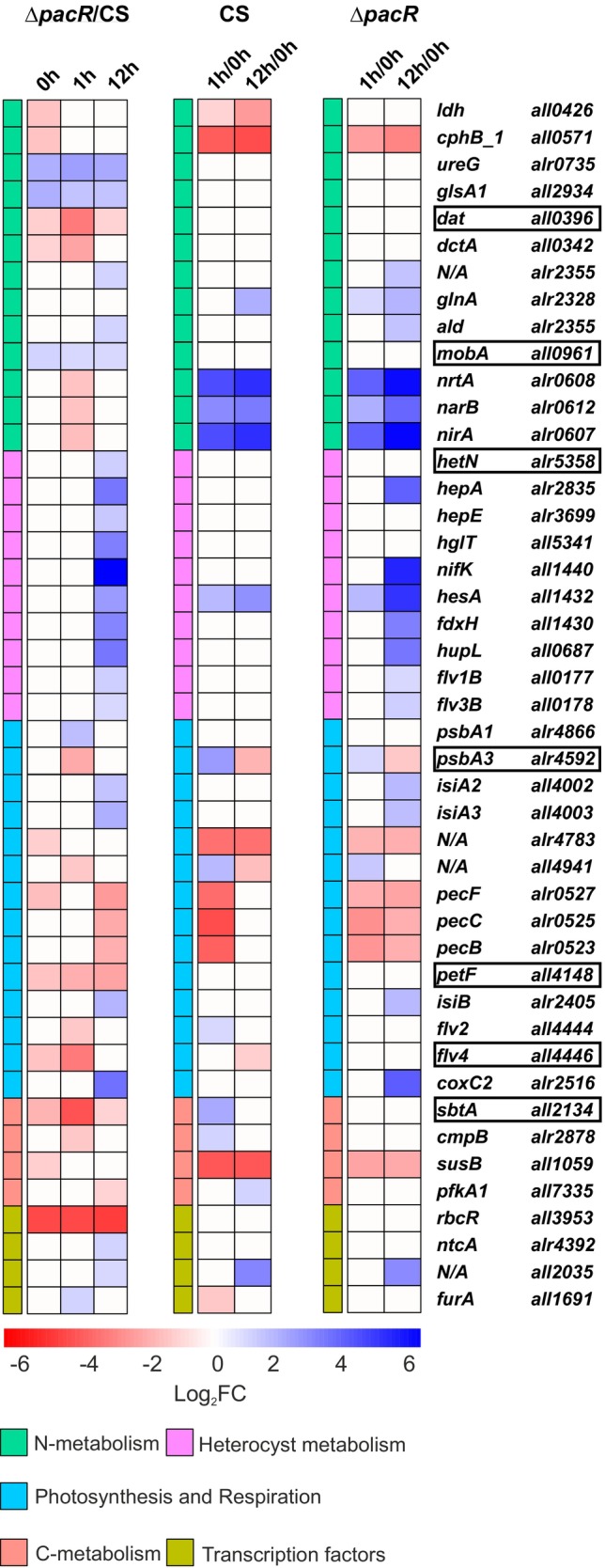
Expression patterns in CS and ∆*pacR* mutant in NH_4_^+^ and during the shift to NO_3_^−^. The selected genes represent central metabolic pathways affected in the mutant. A complete list of annotated affected genes can be found in Tables S4‐S8. **∆*pacR*/CS column:** Differential gene expression between ∆*pacR* and CS at 0 h, 1 h and 12 h after the shift to NO_3_^−^. **CS column:** Differential gene expression in CS between 0 h and 1 h and between 0 h and 12 h. **∆*pacR* column:** Differential gene expression in ∆*pacR* between 0 h and 1 h and between 0 h and 12 h. Differential expression is shown as log_2_FC ≧ I1I, p < 0.05, n = 3 biological replicates per strain and time point. Genes bound by PacR (Picossi et al., 2015) are highlighted in boxes.

The transcription level of PacR (*rbcR*) in the CS remained independent of the nitrogen source, showing no differential expression between timepoints (Figure 5; Tables S7 and S8), as reported previously (Picossi et al., 2015). Strikingly, NtcA (*ntcA*) transcripts were slightly but statistically significantly upregulated at t = 12 h in the Δ*pacR* (Figure 5; Table S6). Additionally, the transcript levels of another global transcriptional regulator, FurA (*furA*), were slightly elevated in the mutant 1 h after the shift, as the transient downregulation observed in the CS was absent in the mutant (Figure 5; Tables S5, S7, and S8). Moreover, no differential expression of CmpR‐transcripts was observed between the CS and the ∆*pacR* mutant.

#### 3.4.2 Nitrogen metabolism

**FIGURE 6:**
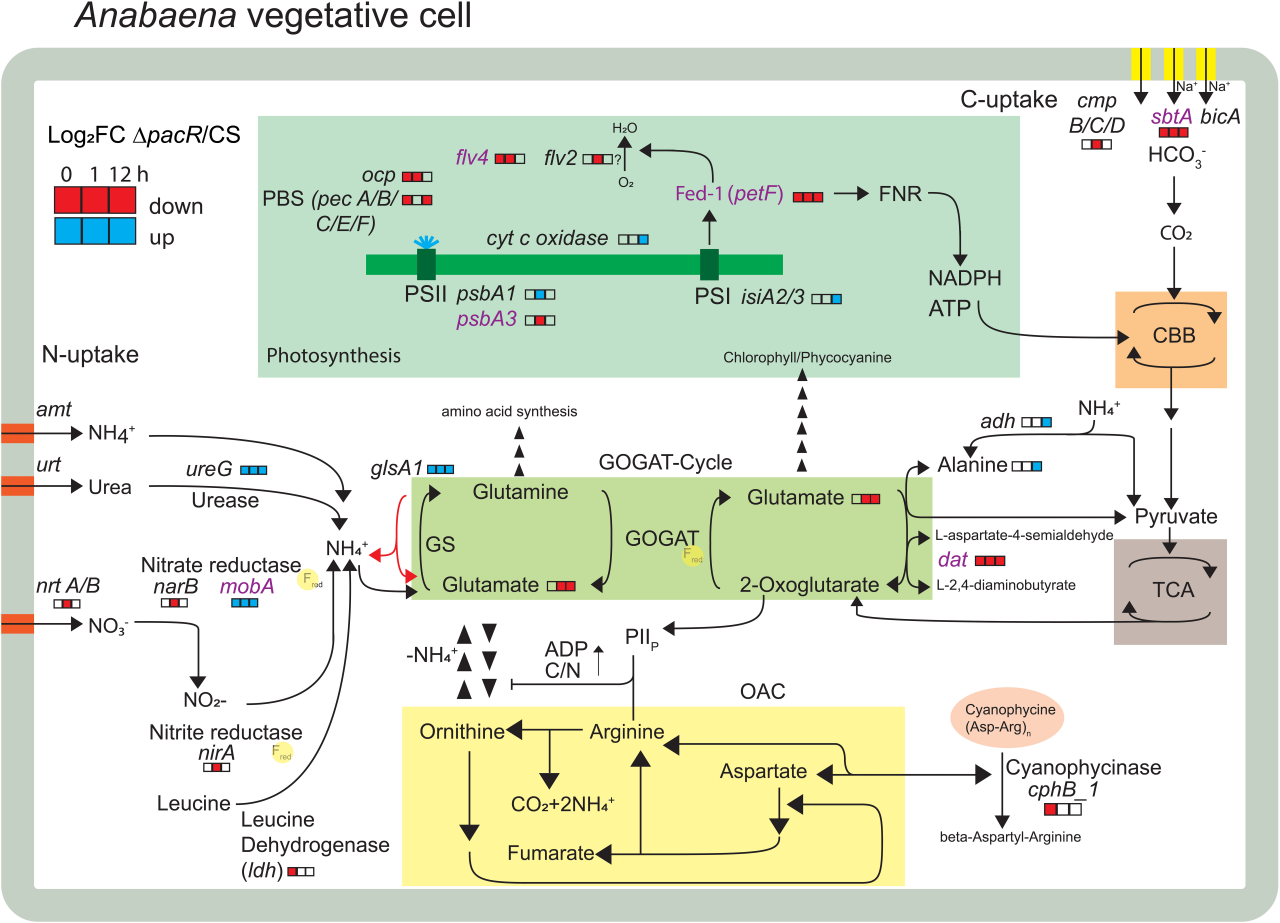
Overview of modified central metabolic pathways in the Δ*pacR* mutant. The schematic illustrates an *Anabaena* vegetative cell, highlighting key pathways involved in C‐ and N‐metabolism, including the GS/GOGAT‐cycle, OAC cycle, cyanophycine synthesis, the CBB cycle and the TCA cycle. The main components of the photosynthetic electron‐transport chain are also depicted. Following nitrogen import through NO_3_^−^ transporters, NO_3_^−^ is reduced to NH_4_^+^ via the action of nitrate (NarB) and nitrite (NirA) reductases; then, ammonium is incorporated into glutamate by ATP‐dependent glutamine synthetase (GS) producing glutamine, whose amido group is transferred to 2‐oxoglutarate (2‐OG) by glutamate synthase (GOGAT). 2‐OG is a product of carbon assimilation. Redox equivalents required for NO₃^−^ reduction and GS/GOGAT enzyme activity are provided by photosynthetic light reactions, which generate reduced ferredoxin (Fed). Fixed nitrogen can be stored as the reserve polymer cyanophycin through the action of a cyanophycin‐synthesizing enzyme. Storage regulation is controlled via a negative feedback loop involving arginine and the PII protein, which responds to ADP levels and the cellular C/N balance. The locations where reduced Fed is required for enzymatic activity are marked with yellow bubbles. Genes or metabolites found to be upregulated (blue) or downregulated (red) in the ∆*pacR* mutant compared to the control strain (CS) are highlighted in colored boxes, both before (t = 0 h) and at 1 h or 12 h after the shift from NH₄^+^ to NO₃^−^ as the N‐source in the medium. Genes with promoters directly bound by PacR (Picossi et al., 2015) are indicated in purple.

The *nirA* operon (*nirA*‐*nrtABCD*‐*narB*), which codes for the NO_3_^−^ uptake machinery (Frías & Flores, 2010), was highly upregulated in the CS 1 h after the shift to NO_3_‐ whereas this upregulation was strikingly delayed in the ∆*pacR* mutant, with a slight but significantly lower transcript level at t = 1 h (Figure 5; Tables S5, S7, and S8). Additionally, *mobA*, a gene required for Mo‐cofactor biosynthesis, which is directly bound by PacR (Picossi et al., 2015), was also found to be constitutively upregulated in Δ*pacR* in this work (Figure 5; Tables S4‐S8). Related to the GS/GOGAT‐cycle, the transcript levels of glutaminase 1 (*glsA1*) (Zhou et al., 2008) was slightly upregulated in ∆*pacR* at all time points (Figure 5; Tables S4‐S6), while the transcript of glutamine synthetase (*glnA*) (Álvarez‐Escribano et al., 2024) was slightly upregulated in ∆*pacR* 1 h after the shift and in both strains 12 h after the shift in relation to t = 0 h (Figure 5; Tables S7 and S8). The transcript levels of *dat* (*all0396*), which encodes diaminobutyrate‐pyruvate transaminase and has a promoter directly bound by PacR (Picossi et al., 2015), were consistently downregulated in ∆*pacR* compared to the CS, showing slight downregulation at both t = 0 h and t = 12 h and intermediate downregulation at t = 1 h (Figure 5; Tables S4‐S6). Leucine dehydrogenase (*ldh*) transcripts were slightly downregulated in ∆*pacR* compared to the CS before the shift. While transcription in the CS showed slight to intermediate downregulation after the shift, no significant changes were observed between time points for the mutant (Figures 5 and 6; Tables S4 and S7). The transcript level of alanine dehydrogenase (*ald*) (Pernil et al., 2010) was slightly upregulated in Δ*pacR* compared to the CS at t = 12 h (Figure 5; Tables S6‐S8). Interestingly, transcription of the urease accessory protein (*ureG*) was constitutively intermediately upregulated in the mutant in relation to the CS (Figures 5 and 6; Tables S4‐S6). Additionally, the cyanophycinase gene *cphB1* was slightly downregulated in ∆*pacR* before the shift (Figures 5 and 6; Table S4).

#### 3.4.3 Heterocyst formation

Many genes associated with heterocyst formation were upregulated in the Δ*pacR* strain 12 h after the shift compared to the CS (Figure 5; Table S6). Genes involved in the formation of the Hep‐layer, such as *hepA* (X. P. Wang et al., 2016; Y. Wang et al., 2007), as well as various glycosyl transferases, like *hepE* (X. P. Wang et al., 2016) were upregulated in various degrees. A glycolipid synthase (*hglT*) involved in Hgl‐layer formation (Zeng & Zhang, 2022) was also intermediately upregulated. Several nitrogenase‐related genes were upregulated, including intermediate to high upregulation of the main *nif*‐gene cluster as well as intermediate upregulation of *nifV*, belonging to the additional *nif*‐gene cluster found in *Anabaena* (Stricker et al., 1997). Concomitantly, the heterocyst‐specific ferredoxin (*fdxB*) (Pils et al., 2004) and the uptake hydrogenase genes (*hupL*, *hupS)* also showed intermediately increased expression. The heterocyst‐specific *flv1B* and *flv3B* transcripts (Ermakova et al., 2013) were slightly upregulated.

#### 3.4.4 Carbon metabolism

The gene (*sbtA*) encoding a putative SbtA carbon transporter orthologue (Herrero & Flores, 2019) was slightly downregulated in Δ*pacR* compared to the CS at t = 0 h and t = 12 h. Unlike the CS, it did not exhibit transient upregulation at t = 1 h, resulting in a more pronounced downregulation in the mutant at this time point (Figure 5; Tables S4‐S8). A similar pattern was observed for *cmpB* transcripts, which encode a bicarbonate transporter (Herrero & Flores, 2019); however, at t = 1 h, *cmpB* expression was only slightly lower in Δ*pacR* than in CS (a similar tendency was observed for the TPM values of the entire *cmpABCD* operon) (Figure 5; Tables S5, S7, S8; Appendix S4). Moreover, sucrose‐synthase (*susB*) transcription (Katoh et al., 2004) was slightly downregulated at t = 0 h in Δ*pacR* compared to the CS, whereas in both strains the gene was downregulated after the shift, albeit only intermediately in the mutant and highly in the CS (Figure 5; Tables S4, S7, S8).

#### 3.4.5 Photosynthesis and respiration

Interestingly, *psbA1* transcripts, encoding the PSII D1 protein (Sicora et al., 2009) were slightly upregulated in the mutant compared to the CS at 1 h after the shift (Figure 5; Table S5). In contrast, the transient upregulation of *psbA3* (Summerfield et al., 2008) at t = 1 h, whose promoter is directly bound by PacR (Picossi et al., 2015), was only slight in Δ*pacR*. In the CS, this upregulation was intermediate, resulting in an intermediate downregulation of this gene at t = 1 h (Figure 5; Tables S5, S7, S8).

Transcripts of *isiA2* and *isiA3*, iron‐stress‐induced proteins with homology to CP43 (Nagao et al., 2021), were slightly upregulated after 12 h in ∆*pacR* compared to the CS, as the upregulation of these genes occurred only in the mutant (Figure 5; Tables S6, S8).

Before the shift, the phycoerythrocyanin (*pec)* operon, which encodes the components of the light‐harvesting phycobilisome (PBS) complex (Gollan et al., 2020), was slightly downregulated in ∆*pacR* (significantly for *pecE* and *pecF*). Genes of this operon were then downregulated to about the same level in both strains at t = 1 h and were then significantly expressed at slightly to intermediately higher levels in the CS compared to Δ*pacR* after 12 h (Figure 5; Tables S4‐S8). The transcript level of *all4940* and *alr4783* associated with the orange carotenoid protein (OCP) (López‐Igual et al., 2016) were slightly downregulated in ∆*pacR* compared to the CS at t = 0 h and then downregulated significantly to similar levels in both strains after the shift (Figure 5; Tables S4, S7, S8).

The following transcripts of cytochrome c oxidase subunits were intermediately to highly upregulated at t = 12 h in ∆*pacR* compared to the CS: *coxA2* and *coxC2* corresponding to COX2 (homologous to aa3‐type cytochrome c oxidases) as well as *coxA3*, corresponding to COX3 (homologous to alternative respiratory terminal oxidases) (Figure 5; Tables S6, S8). Both COX2 and COX3 were previously found to be upregulated under nitrogen deficiency (Wünschiers et al., 2006). The transcript levels of vegetative cell‐specific Fed‐1 (*petF*) (Schmitz & Böhme, 1995) were constitutively downregulated in Δ*pacR*, ranging from slight downregulation at early time points (t = 0 h and t = 1 h) to intermediate levels at t = 12 h (Figure 5; Tables S4‐S6). This downregulation may be attributed to PacR binding to the *petF* promoter. There was slightly lower expression of *flv2* at *t* = 1 h compared to the CS. Additionally, at the same time point, *flv4* was intermediately downregulated relative to the CS (Figures 5 and 6; Tables S5, S7). The expression of *flv1A* (*all3891*) and *flv3A* (*all3895*) (Ermakova et al., 2013) did not differ significantly between the mutant and the CS. Their expression kinetics followed those of the CS, showing a non‐significant transient increase 1 h after the shift, which was, however, less pronounced in the mutant based on TPM values (Appendix S4).

## 4 DISCUSSION

In this study, we found that the *∆pacR* mutant of *Anabaena* forms heterocysts, resembling diazotrophic growth, even under nitrogen‐replete conditions. This heterocyst formation occurs following a shift in the nitrogen source from NH_4_^+^ to NO_3_^−^ in the growth medium (Figure 2A, B; Table S1). This is consistent with the findings of a parallel study by Lin et al. (2025), which reported a higher heterocyst frequency in the Δ*pacR* mutant following the same nutrient shift. *Anabaena* typically undergoes heterocyst differentiation within the first 24 h after N step‐down (Xing et al., 2022). To gain insights into the cellular changes preceding heterocyst formation, we collected metabolic and RNA‐seq data at early time points (0 h, 1 h, 12 h). Additionally, physiological measurements were performed 48 h after the shift, when heterocyst formation was complete, and the growth phenotype became apparent (Figures 1, S1A, and S2A). A similar growth phenotype was also observed by Lin et al. (2025). The Δ*pacR* mutant exhibited upregulation of genes associated with heterocysts, not constitutively, but specifically during the 12 h interval following the shift in the nitrogen source from NH_4_^+^ to NO_3_^−^ (Figures 5 and 6; Tables S6, S8). These findings suggest that the deletion of PacR does not directly trigger heterocyst differentiation; instead, the response seems to be an effect of the shift from NH_4_^+^ to NO_3_^−^, which likely disrupts the cellular C/N balance (Zhang et al., 2018). Indeed, the Δ*pacR* mutant exhibited a significantly elevated C/N ratio (Figure 2 D; Table S2), indicating intracellular nitrogen limitation. The Δ*pacR* mutant indeed shows impaired NO_3_^−^ uptake (Figures 2C and S3), accompanied by delayed induction of *nirA* operon transcripts following the shift to NO_3_^−^ containing medium (Figures 5 and 6; Tables S6‐S8). This could reflect a low abundance of NO_3_^−^ transporters in the membrane and a lower abundance of NirA and NarB proteins, and thus a reduced capacity for NO_3_^−^ uptake. Although the transcript level of the *nirA* operon aligns with that of the CS at 12 h, the protein levels may not follow the same pattern, potentially contributing to persistently low NO_3_^−^ uptake. Indeed, despite comparable transcript levels of transporters and nitrate and nitrite reductases in the mutant and CS at 12 h, impaired NO_3_^−^ uptake in the mutant has been observed throughout the entire 120 h duration of the experiment. Interestingly, Lin et al. (2025) reported a similar impairment of nitrate uptake and *nirA* expression, and further confirmed the direct binding of PacR to both *nirA* and *ntcB*.

Although direct regulation of the *nir* operon cannot be inferred from the present data, a potential role in the modulation of nitrate and nitrite reductases by molybdenum cofactor biosynthesis remains possible. Nitrate reductases are molybdoenzymes carrying a molybdenum cofactor. Mo‐cofactor biosynthesis requires several genes (Puerta‐Fernández & Vioque, 2011), among which the *mobA* gene was found to be constitutively upregulated in the mutant, regardless of the nitrogen source (Figures 5 and 6) and was also found to be directly bound by PacR (Picossi et al. 2015). This may suggest an involvement of PacR in modulating the *nir* operon via *mobA* regulation.

A potential limitation in reducing equivalents from photosynthetic light reactions is indicated by the constitutive downregulation of the *petF* transcript, which encodes the vegetative cell‐specific Fed‐1, in the ∆*pacR* mutant (Figures 5 and 6; Tables S4‐S6). PacR, which directly binds to the *petF* promoter (Picossi et al., 2015), could directly influence *petF* expression, likely resulting in reduced Fed‐1 levels. Since the functions of nitrite reductase (NirA) and nitrate reductase (NarB) rely on vegetative‐specific Fed‐1, the decrease in *petF* levels may impair both NO_3_^−^ uptake and the conversion of NO_3_^−^ to NH_4_^+^ (Watzer et al., 2019). Indeed, our data show a slight but statistically significant decrease in the size of the photo‐reducible Fed‐pool (Figure S5A, B), suggesting that impaired electron flux from Fed may contribute to hindered nitrate assimilation.

The depletion of free amino acid pools is typical under nitrogen‐depleted conditions in *Anabaena* (Perin et al., 2021), indicating a lack of nitrogen for metabolic functions. Here, the reduced glutamate levels in the mutant after the shift compared to the CS (and non‐significant decrease in glutamine, and 2‐OG) suggests an imbalance in C/N metabolism (Figures 4 and S6). However, this is in contrast to the usual response in wild‐type *Anabaena*, where glutamate levels rise and glutamine levels drop when shifting to diazotrophic conditions (Perin et al., 2021). Since glutamate and glutamine are crucial indicators of nitrogen status, this reduction implies a weakened nitrogen assimilation capacity and a reduction in GS/GOGAT‐cycle activity.

2‐OG, which serves as a signaling molecule for the C/N balance (Robles‐Rengel et al., 2019), is known to increase following nitrogen deprivation, triggering heterocyst formation in *Anabaena* (Laurent et al., 2005; Muro‐Pastor et al., 2001; Zeng & Zhang, 2022). In contrast, the ∆*pacR* mutant demonstrated low 2‐OG levels (Figures S6 and S7) and showed no significant differences in the 2‐OG/Glu ratios (Figure S8) despite the occurrence of heterocyst formation.

Changes in metabolite levels in the GS/GOGAT‐cycle may be an indirect consequence of PacR‐deletion, potentially due to the constitutive upregulation of *glsA1* transcripts encoding phosphate‐dependent glutaminase 1 (Figures 5 and 6; Tables S4‐S6). The transcripts of glutaminase 1, primarily responsible for glutamine deamination, are typically downregulated transiently under nitrogen‐limiting conditions in wild type (Zhou et al., 2008). Elevated glutaminase activity could impair the function of ATP‐dependent glutamine synthetase (GS, *glnA*), a key regulatory enzyme in the GS/GOGAT cycle. *glnA* is typically expressed at higher levels in the absence of combined nitrogen and is regulated by NtcA (Flores et al., 2019). Also, the GS has been shown to be active under combined nitrogen‐limited conditions (Forchhammer & Selim, 2020; Galmozzi et al., 2010) and shows increased activity when N_2_ is used as the nitrogen source (Flores & Herrero, 1994; Zhou et al., 2008). Notably, inhibiting GS with the specific inhibitor MSX can counteract the suppression of nitrogen fixation and heterocyst formation by fixed nitrogen in *Anabaena* sp., which indicates that NH_4_^+^ assimilation via GS is required for NH_4_^+^ repression of the uptake of alternative nitrogen sources (Flores & Herrero, 1994; Mishra, 2003). A similar effect may occur in Δ*pacR* due to potential glutamine degradation via glutaminase 1. Interestingly, *glnA* is upregulated in the mutant after just 1 h, whereas no such response is observed in the CS, suggesting a compensatory response to putatively increased glutaminase activity and suggesting that NtcA‐controlled pathways are activated in the mutant. The balance between glutamine synthesis and degradation may determine whether a glutamine shortage occurs, ultimately affecting nitrogen availability for cellular metabolism.

Alanine levels were significantly higher in the Δ*pacR* mutant compared to the CS following the 12 h shift to NO_3_^−^ (Figures 4 and S6). An increase in alanine levels following the shift to diazotrophic conditions was previously observed in *Anabaena* wild type (Perin et al., 2021) and *Anabaena cylindrica* (Rowell & Stewart, 1976) since alanine is needed for carbon transfer to heterocysts (Burnat et al., 2014; Pernil et al., 2010). Therefore, the elevated alanine levels in this study signify a metabolic state in the Δ*pacR* mutant that resembles diazotrophic conditions, potentially linking them to heterocyst formation. In *Anabaena spp*., the gene coding for alanine dehydrogenase (ADH), *ald*, is either exclusively expressed or exhibits higher expression levels following the shift to diazotrophic conditions (Pernil et al., 2010). Furthermore, ADH activity has been shown to increase under nitrogen deficiency in *Anabaena cylindrica* (Rowell & Stewart, 1976). These observations indicate that the upregulation of *ald* in Δ*pacR* may lead to higher levels of active enzyme and reflect a metabolic adaptation consistent with diazotrophic conditions. Interestingly, the ADH pathway also possesses synthetic activity, converting pyruvate and NH_4_^+^ into alanine, which facilitates NH_4_^+^ assimilation under conditions of GS/GOGAT‐cycle inactivation (Flores & Herrero, 1994; Meeks et al., 1977). In this study, the GS/GOGAT‐cycle impairment is likely due to reduced GS efficiency and previous research indicates that NH_4_^+^ incorporation through ADH increases when GS is inactivated by MSX treatment (Meeks et al., 1977). This suggests that there may be increased synthetic ADH activity in the mutant, which may contribute to the increased alanine levels and provide a compensatory mechanism that serves to counterbalance decreased operation of the GS/GOGAT‐cycle.

Diaminobutyrate‐pyruvate transaminase *(dat*), which catalyzes the transamination of L‐2,4‐diaminobutyrate to 2‐OG, forming L‐aspartate–semialdehyde and glutamate was constitutively downregulated (Figures 5 and 6; Tables S4‐S6). Moreover, L‐2,4‐diaminobutyrate also links to arginine biosynthesis, as L‐aspartate‐4‐semialdehyde is derived from L‐aspartate. This suggests that its activity may be functionally connected to the GS/GOGAT cycle, protein synthesis and nitrogen storage. Moreover, leucine dehydrogenase (*ldh*) transcripts remained consistently low in the mutant despite the absence of PacR binding to its promoter (Figures 5 and 6; Tables S4, S7). In contrast, *ldh* was highly expressed in the CS before the shift and subsequently downregulated. This pattern suggests that PacR‐mediated regulation of transaminases may help buffer nitrogen metabolism during short‐term fluctuations. By redistributing nitrogen through transaminase activity, the cell may prevent unnecessary heterocyst formation in response to transient changes in external nitrogen levels.

Importantly, the transcript level of urease accessory protein (UreG), *ureG*, is constitutively upregulated in the mutant (Figures 5 and 6; Tables S4‐S6), which may suggest a direct involvement of PacR. Also, cyanophycinase B1 (*cphB1*) transcript is downregulated in the mutant already in the presence of NH_4_^+^ before shifting the cells to nitrate (Figures 5 and 6; Tables S4, S7, S8). This suggests that the broader nitrogen metabolism, including the uptake of alternative nitrogen sources and nitrogen storage via the OAC, is affected by PacR‐deletion. The upregulation of urease‐related genes may also indicate a predisposition of the mutant to release NH_4_^+^ from nitrogen‐containing metabolites. However, it is not known whether PacR directly binds to these genes, leaving their direct involvement in this nitrogen fixation pathway uncertain.

Taken together, these results suggest that inefficiency in the GS/GOGAT‐cycle may reduce the mutant's ability to fix NH_4_^+^ intracellularly into metabolically available forms. Moreover, since transaminases and urease may be affected, the dissemination of NH_4_^+^ may be impaired across multiple metabolic processes, which also seems to be indicated by higher alanine levels and *ald* upregulation. By directly regulating *sbtA* transcription and influencing *cmpB* expression (Figures 5 and 6; Tables S4‐S8), PacR helps adjust carbon intake in response to the C/N balance. In alignment with our results, Bolay et al. (2022) found that a knockdown‐mutant of the PacR‐homologue in *Synechocystis* sp. PCC6803 also displayed diminished *sbtA* expression, confirming the central role of PacR in regulating carbon metabolism in cyanobacteria. Chip‐Seq analysis by Picossi et al. (2015) confirms that PacR may directly regulate three C‐uptake systems under inorganic carbon limitations: the bicarbonate transporter *bicA* and two CO_2_ uptake genes (*ndhF3* and *ndhF4*). Recently, Lin et al. (2025) demonstrated under ambient CO_2_ conditions that the promotors of *cmpA*, *bicA* and *sbtA* (all encoding bicarbonate transporters) are directly bound by PacR. Their results suggest that PacR activates *bicA* and *sbtA* in NH_4_^+^‐containing medium, slightly represses them in NO_3_^−^‐containing medium and represses *cmpA* irrespective of the N‐source.

The observed decrease in PSI yield in the ∆*pacR* mutant is mainly due to increased acceptor‐side limitation (Figure 3A, B) linked to significantly decreased O_2_ photoreduction and a smaller pool of photo‐reducible Fed (Figures 3C, D and S5C; Table S3). Since O_2_ photoreduction is mainly attributed to Flv proteins (Santana‐Sánchez et al., 2023), the downregulation of *flv2* and *flv4* transcripts in the mutant (Figures 5 and 6; Tables S4, S5, S7, S8) along with previous findings that PacR binds to the promoter regions of *flv1A* and *flv4* (Picossi et al., 2015) indicates that these regulatory changes likely contribute to the observed impairment.

In conclusion, deletion of the global transcription factor PacR in *Anabaena* induces heterocyst formation even in NO₃^−^‐containing medium, likely due to reduced NO_3_^−^ uptake, disrupted NH_4_^+^ assimilation in the GS/GOGAT cycle and intercellular C/N unbalance. This phenotype may result from PacR's regulatory influence on key genes involved in nitrogen and carbon fixation, as well as photosynthetic performance. Our data suggests that a shortage of reducing equivalents is one of the contributing factors to impaired nitrogen metabolism. These findings highlight PacR's crucial role in coordinating photosynthesis, nitrogen and carbon metabolism.

## AUTHOR CONTRIBUTIONS

YA conceptualized the project. EW performed all experiments and wrote the first draft of the manuscript. YA, TH, ASS, and LN supervised the experiments. All authors contributed to editing and proofreading. All authors have read and approved the final manuscript.

## FUNDING INFORMATION

This work was supported by the NovoNordisk Foundation (PhotoCat, project # NNF20OC0064371 to YA), by the Jane and Aatos Erkko Foundation (PhotoFactory project, to YA). EW and TH were partially supported by the University of Turku Graduate School (UTUGS) and by the Turku Collegium for Science, Medicine and Technology, respectively.

## Supporting information


**Data S1.** Supporting Information

**Appendix S1.** RNA‐seq_DEanalysis_dpacRvsCS

**Appendix S2.** RNA‐seq_DEanalysis_CSvsCS_dpacRvsdpacR

**Appendix S3.** RNA‐seq_Enrichment Analysis_dpacRvsCS

**Appendix S4.** RNA‐seq_TPM values

## Data Availability

The RNA sequencing data used for Figures 5 and 6, Tables S4‐S8 and Appendix [Link], [Link] is available in NCBI under the GEO accession number GSE280386. Data supporting the findings of this study are available in the manuscript or supplementary materials. Microscopic images and physiological source data are available upon request.
