# Supplementary material for: The role of the LysR‐type transcription factor PacR in regulating nitrogen metabolism in Anabaena sp. PCC7120
Source: Physiol Plant. 2025 May 5;177(3):e70248. doi: 10.1111/ppl.70248 (PMC12052932; doi:10.1111/ppl.70248)
Supplement: Supplementary file 1 — Data S1. Supporting Information [file PPL-177-e70248-s005.pdf]

SUPPLEMENTAL

The role of the LysR-type transcription factor PacR in regulating nitrogen metabolism in *Anabaena* sp. PCC7120

E. Werner [1], T. Huokko [1], A. Santana-Sanchez [1], S. Picossi[2], L. Nikkanen[1], A. Herrero[2], Y. Allahverdiyeva[1,*]

[1] Molecular Plant Biology, Department of Life Technologies, University of Turku, Turku FI- 20014, Finland

[2] Instituto de Bioquímica Vegetal y Fotosíntesis, Consejo Superior de Investigaciones Científicas, Universidad de Sevilla, Seville E-41092, Spain

***Correspondance:**

Yagut Allahverdiyeva, E-mail: allahve@utu.fi

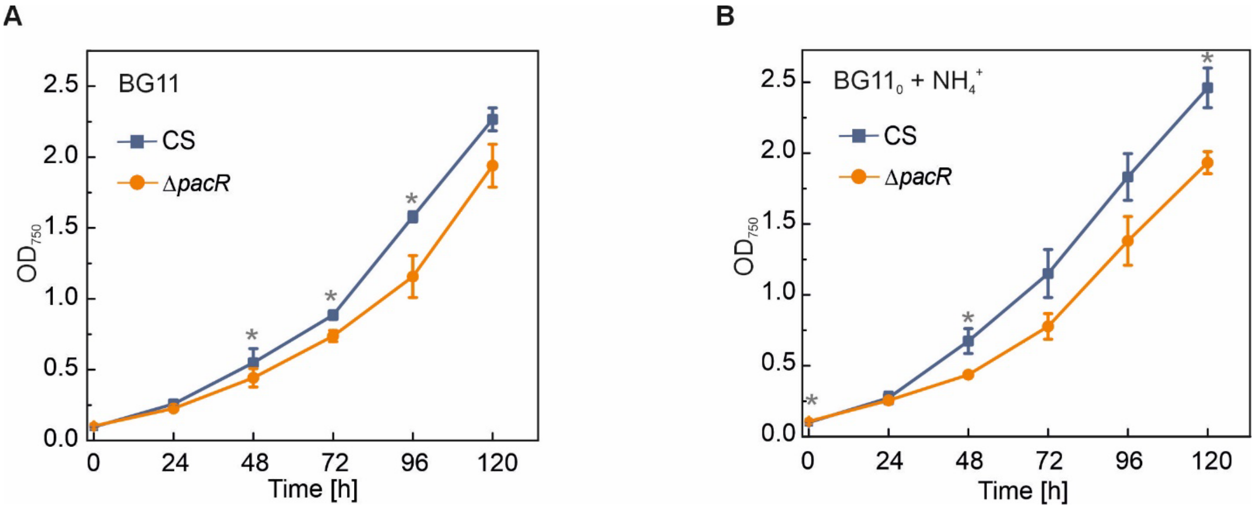

Figure S1. Growth phenotype of the Δ*pacR* mutant and the CS after the shift to NO_3^- or 3 mM NH_4^+ (supplemented on days 1, 2 and 3). Values are means ± SD; *n*= 3 biologically independent experiments; statistically significant differences ($p < 0.05$) compared to the CS are indicated by an asterisk. Statistical analysis was performed using a two-tailed Type 2 t-test.

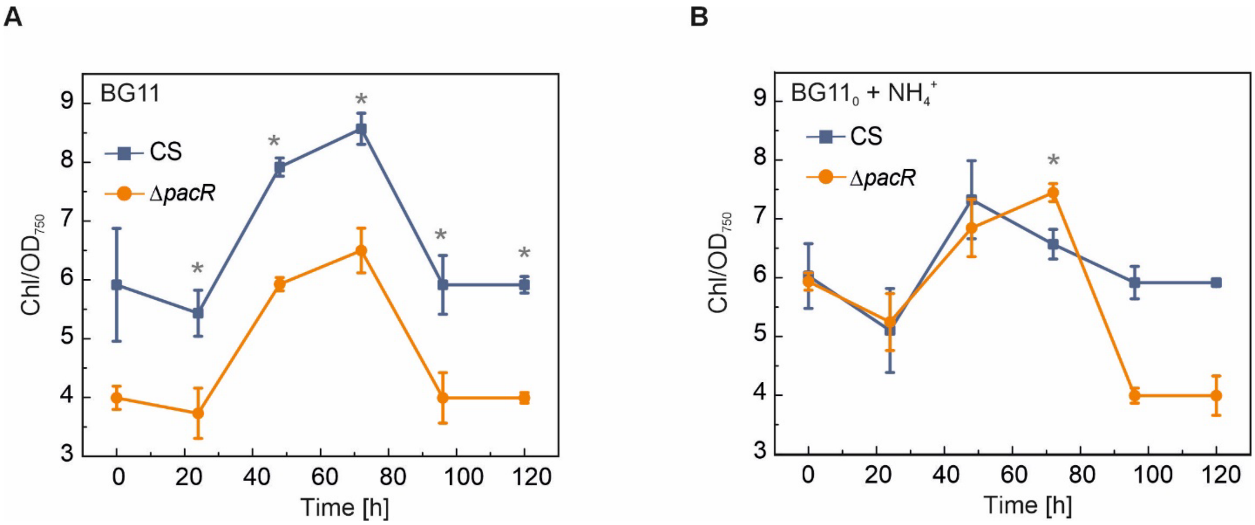

Figure S2. Chl/OD_{750} ratios of the Δ*pacR* mutant and the CS after the shift to NO_3^- or 3 mM NH_4^+ (supplemented on days 1, 2 and 3). **A.** with NO_3^- ; **B.** with NH_4^+. Values are means ± SD; *n*= 3 biologically independent experiments; statistically significant differences ($p < 0.05$) compared to the CS are indicated by an asterisk. Statistical analysis was performed using a two-tailed Type 2 t-test.

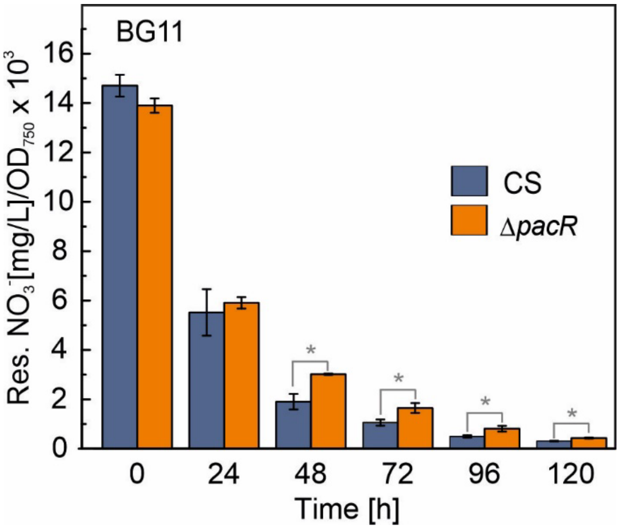

Figure S3. Residual NO_3^- in the growth medium at different timepoints after the shift to NO_3^-. Values are means ± SD; *n*= 3 biologically independent experiments; statistically significant differences ($p < 0.05$) compared to the CS are indicated by an asterisk. Statistical analysis was performed using a two-tailed Type 2 t-test.

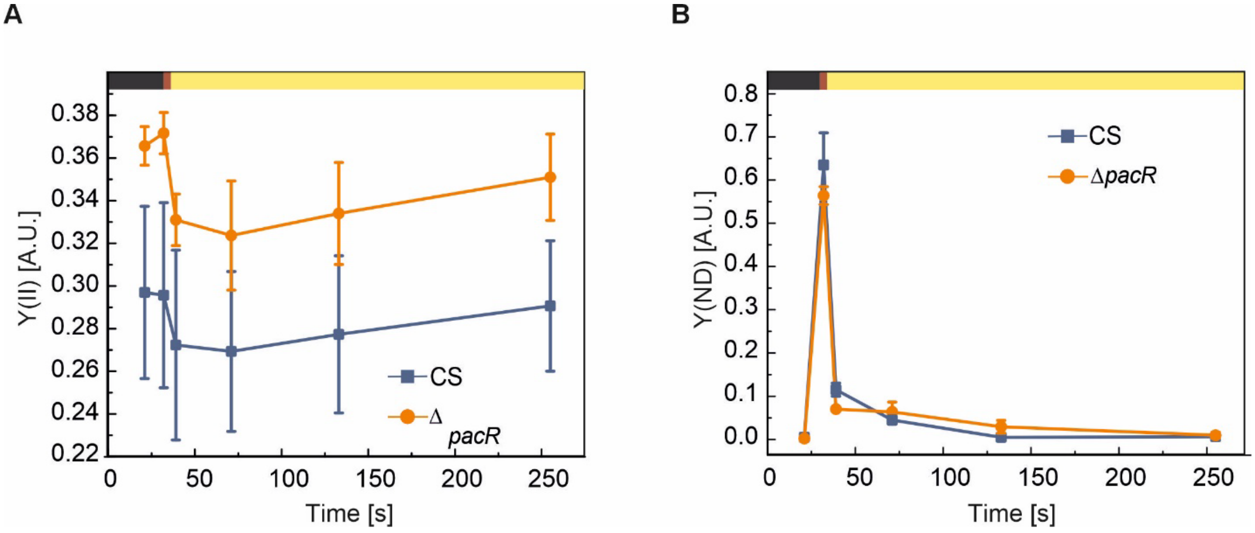

Figure S4. Photosynthetic performance of the Δ*pacR* mutant and the CS 48 h after the shift to NO_3^-. **A.** Effective yield of PSII [Y(II)]; **B.** Donor side limitation of PSI [Y(ND)]. grey bar: darkness, red bar: far red light, yellow bar: actinic light. Values are means ± SD; *n*= 3 biologically independent experiments; statistically significant differences ($p < 0.05$) compared to the CS are indicated by an asterisk. Statistical analysis was performed using a two-tailed Type 2 t-test.

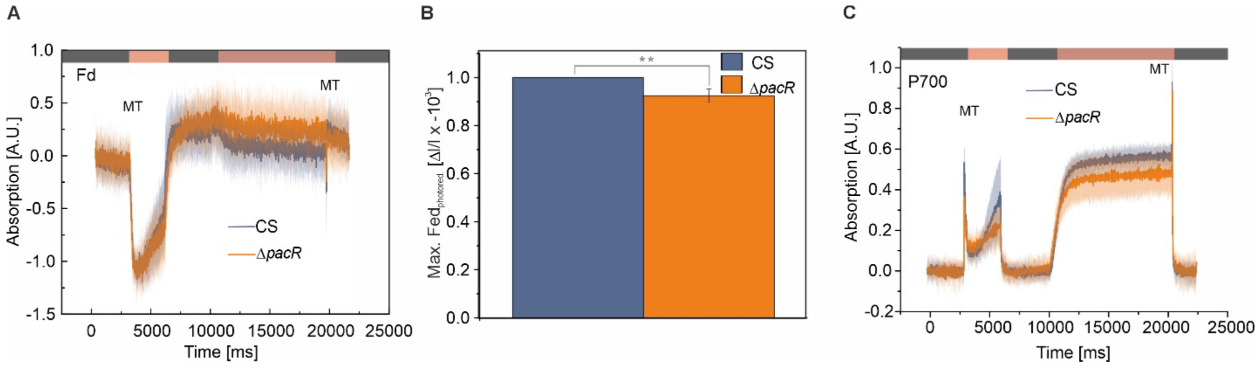

Figure S5. Redox changes of ferredoxin (Fed) and P700 during dark-to-high irradiance transitions of the Δ*pacR* mutant and the CS 48 h after the shift to NO_3^-. **A.** Redox-state of Fed; **B.** Size of the photo-reducible ferredoxin pool. **C.** Redox-state of P700. Grey bar: darkness, dark red bar: far red light, light red bar: red actinic light. MT=multiple turnover flash. Absorbance differences correspond to different redox-states. Values are means ± SD; *n*= 5 biologically independent experiments; statistically significant differences ($p < 0.05$) compared to the CS are indicated by an asterisk. Statistical analysis was performed using a two-tailed Type 2 t-test.

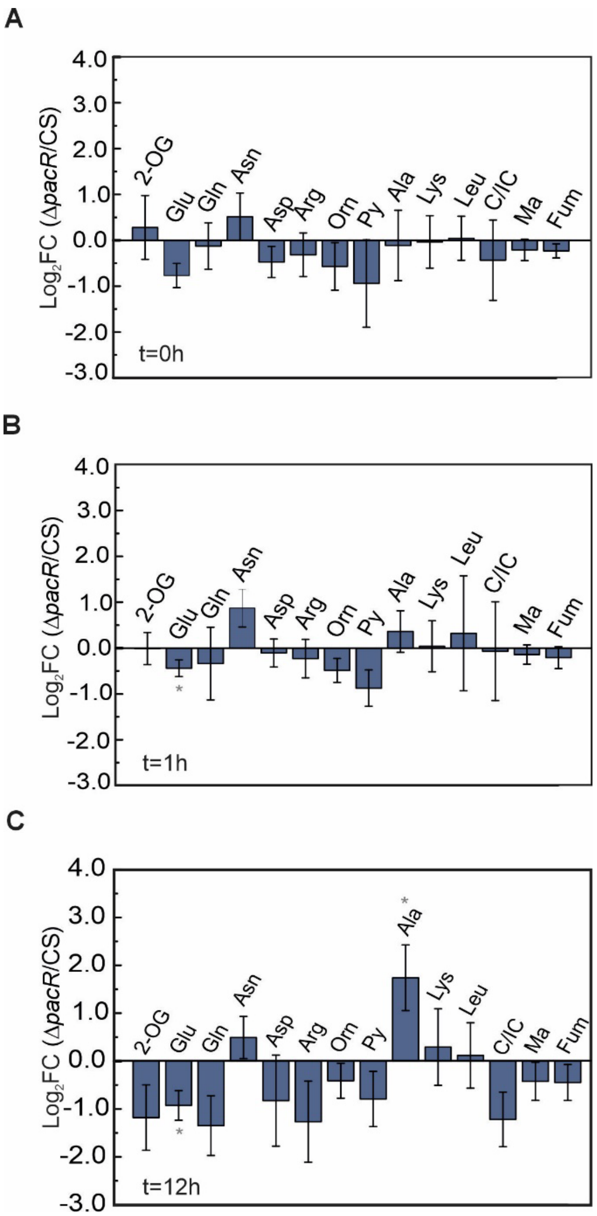

Figure S6. Metabolic analysis of the Δ*pacR* mutant before and 1 h and 12 h after the shift to NO_3^-. **A.** Metabolite levels of Δ*pacR* relative to the control strain (CS) at t= 0 h; **B.** Metabolite levels of Δ*pacR* relative to CS at t= 1 h; **C.** Metabolite levels of Δ*pacR* relative to CS at t= 12 h. 2-OG= 2-Oxoglutarate, Glu= Glutamate, Gln= Glutamine, Asn= Asparagine, Asp= Aspartic acid, Arg= Arginine, Orn= Ornithine, Py= Pyruvate, Ala= Alanine, Lys= Lysine, Leu= Leucine, CI/C= Citrate/Isocitrate, Ma= Malic acid, Fum= Fumarate. Values are mean ratios of ion counts shown as $\log_2$FC ± SD; *n*= 3-6 biologically independent experiments; statistically significant differences ($p < 0.05$) compared to the CS were calculated based on the normalized total ion count and are indicated by an asterisk. Statistical analysis was performed using a two-tailed Type 2 t-test.

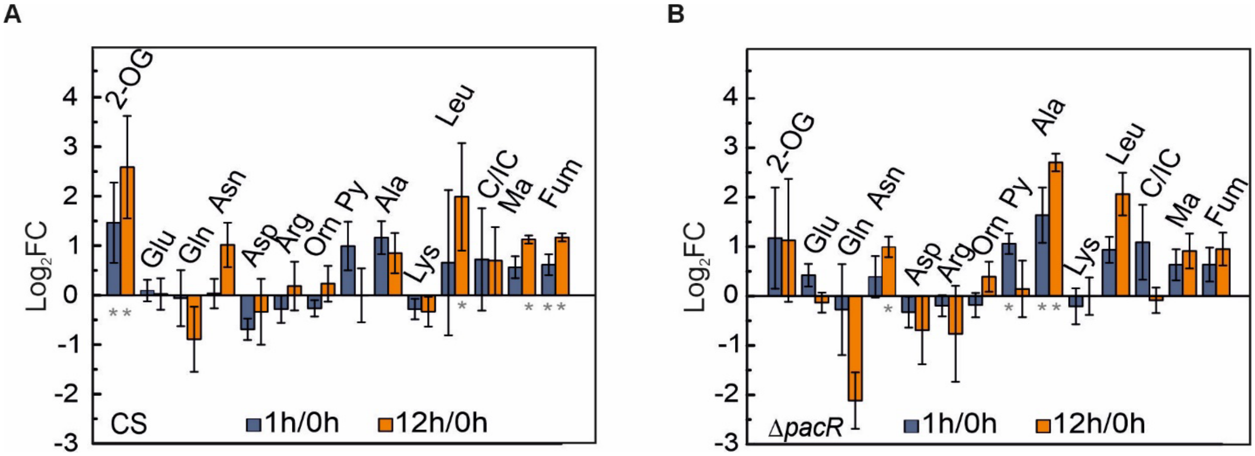

Figure S7. Metabolite levels in the CS and Δ*pacR* mutant before and 1h/12h after the shift to NO_3^-. **A.** CS; **B.** Δ*pacR*. Values of ion count/total ion count for t= 1 h and t= 12 h are in relation to t= 0 h. 2-OG= 2-Oxoglutarate, Glu= Glutamate, Gln= Glutamine, Asn= Asparagine, Asp= Aspartic acid, Arg= Arginine, Orn= Ornithine, Py= Pyruvate, Ala= Alanine, Lys= Lysine, Leu= Leucine, CI/C= Citrate/Isocitrate, Ma= Malic acid, Fum= Fumarate. Values are mean ratios of ion counts shown as log_2FC ± SD; *n*= 3-6 biologically independent experiments; statistically significance differences ($p < 0.05$) compared to t= 0 h were calculated based on the normalized total ion count and are indicated by an asterisk. Statistical analysis was performed using a two-tailed Type 2 t-test.

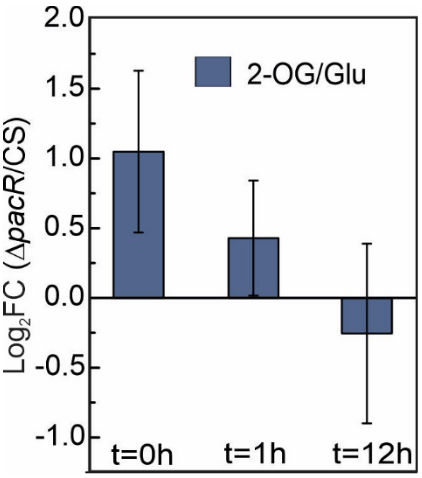

Figure S8. Metabolite levels in the CS and Δ*pacR* mutant before and 1 h and 12 h after the shift to NO_3^-. The log_2FC between 2-OG/Glu ratios between strains are shown for the different timepoints. 2-OG= 2-Oxoglutarate, Glu= Glutamate. Values are means ± SD; *n*= 3 biologically independent experiments; statistically significant differences ($p < 0.05$) compared to the CS are indicated by an asterisk. Statistical analysis was performed using a two-tailed Type 2 t-test.

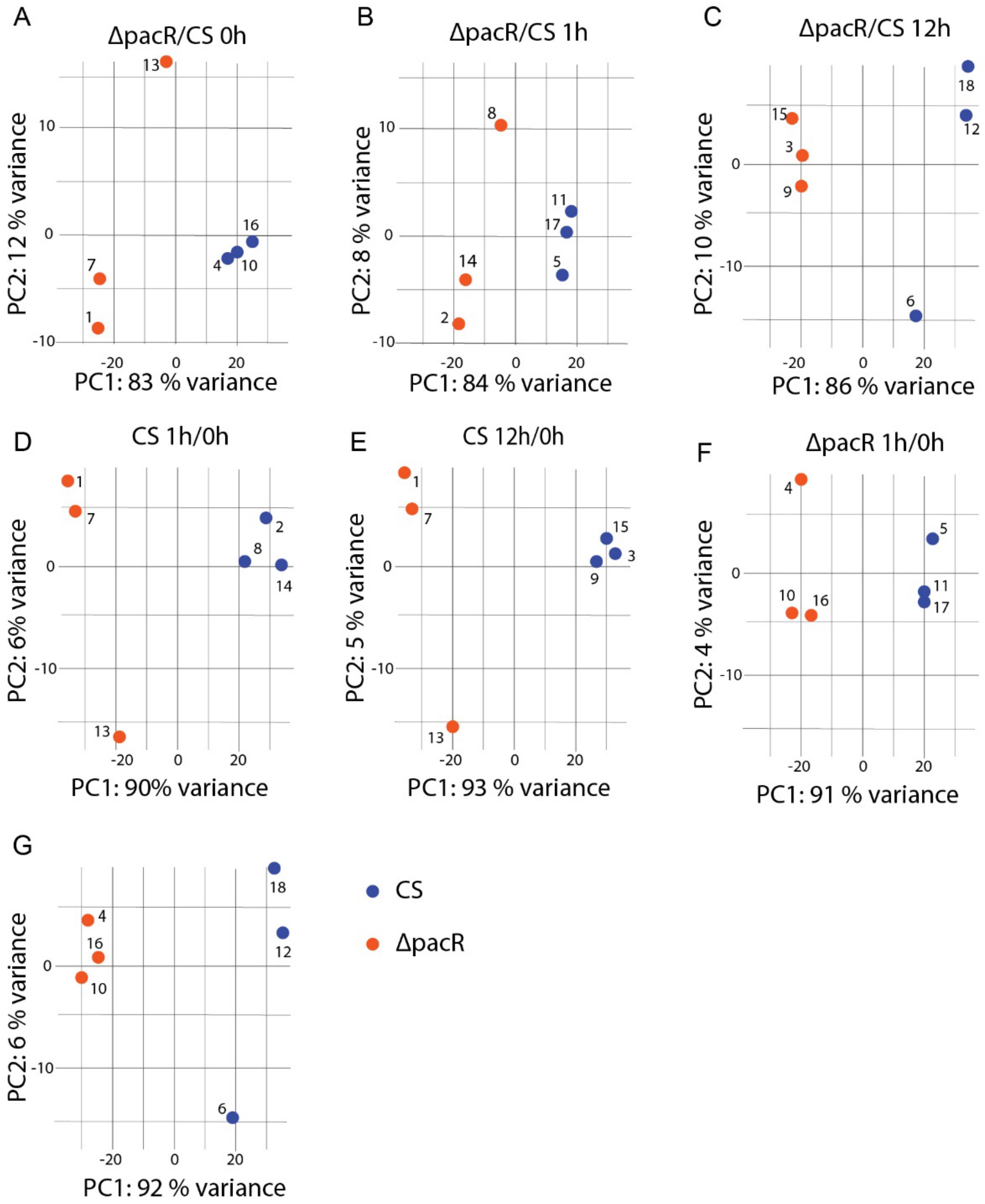

Figure S9. PCA plots generated via the DESeq2 Bioconductor package for experimental level quality control of the RNA-seq data. 3 biological replicates were measured per strain and timepoint. **A.**, **B.** and **C.** show PCA plots for differential expression analysis for Δ*pacR* in relation to the CS. **D.** and **E.** show PCA plots for the differential expression analysis of the CS for timepoints 1 h or 12 h in relation to 0 h. **F.** and **G.** show PCA plots for the differential expression analysis of Δ*pacR* for timepoints 1 h or 12 h in relation to 0 h. The samples are numbered as follows: 1,7,13: 3 biological replicates for the CS at timepoint 0 h; 4,10,16: 3 biological replicates for Δ*pacR* at timepoint 0 h; 2,8,14: 3 biological replicates for the CS at timepoint 1 h; 5,11,17: 3 biological replicates for Δ*pacR* at timepoint 1 h; 3,9,15: 3 biological replicates for the CS at timepoint 12 h; 6,12,18: 3 biological replicates for Δ*pacR* at timepoint 12 h.

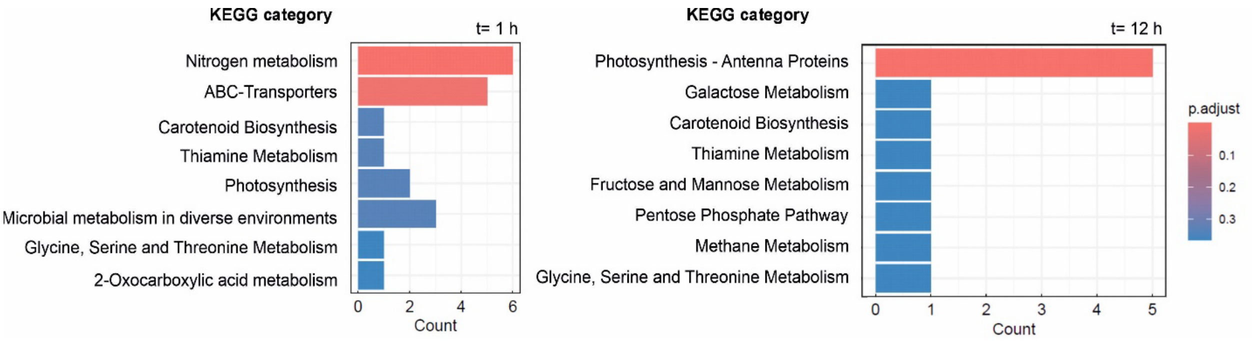

Figure S10. Enrichment Analysis of RNA-seq data. KEGG-pathways that were found to be enriched. P.adjust = adjusted p-value, count = number of genes in category.

Table S1. Heterocyst frequency for the CS and Δ*pacR* strains 48 h after the shift to NO_3^- or 3 mM NH_4^+ (not supplemented on days 1,2 and 3).

**strain**	**nitrogen source of experimental culture**	**total # of cells counted**	**# of vegetative cells**	**# of heterocysts**	**heterocyst frequency [% of total cells counted]**
CS	NH_4^+	4379	4377	2	0.05±0.04
	NO_3^-	5709	5655	54	0.95±0.16
Δ*pacR*	NH_4^+	3754	3744	10	0.26±0.36(1)
	NO_3^-	5102	4722	380	7.45±1.68

(1)higher error due to occurrence of some heterocysts only in one biological replicate

Table S2. N and C content of the CS and Δ*pacR* strains 48 h after the shift to NO_3^-. Values are means ± SD; *n*= 3 biologically independent experiments.

strain	Nitrogen [weight %]	Carbon [weight %]
CS	11.9±0.2	49.3±0.2
Δ*pacR*	10.2±0.8	47.6±0.8

Table S3. The rates of CO_2 and O_2 exchange in the CS and Δ*pacR* mutant 48 h after the shift to NO_3^-. Gas exchange rates are presented as µmol mg Chl a-1 h-1 and values are mean ± SD, n =3 biological replicates. Asterisks indicate statistically significant differences compared to the CS ($p < 0.05$). Statistical analysis was performed using a two-tailed Type 2 t-test.

	**Dark Respiration**	**Gross O_2 evolution (steady-state)**	**Light-induced O_2 uptake (max. peak/steady-state)**	**Net O_2 evolution (steady state)**	**CO_2 uptake (max. peak / steady - state)**
CS	8.9±0.9	173.9±29.6	40.9±3.9*/ 23.1±2.7*	142.1±26.1	342.7±71.4/ 149.6±50.2
Δ*pacR*	13.5±3.7	179.3±12.5	13.0±3.0* / 7.3±5.0*	159.4±11.1	424.6±36.7/ 173.5±34.9

Table S4. Differentially expressed genes at t= 0 h, log_2FC (ΔpacR/CS). Genes marked in thick lines are directly bound by PacR according to Picossi et al. 2015.

**Category**	**ID**	**ENSEMBL_ID**	**Cyanobase_ID**	**log_2FC (ΔpacR/CS)**
**N-metabolism**				
Leucine dehydrogenase	*ldh*	*ENSB:DCJLnD1-vRm8aTd*	*all0426*	-1.45
Cyanophycinase	*cphB_1*	*ENSB:qnAFH-zZi1_Cg7h*	*all0571*	-1.44
Urease accessory protein	*ureG*	*ENSB:thD7sRcdm3nPz9H*	*alr0735*	2.16
Glutaminase 1	*glsA1*	*ENSB:fjcIlBqee15rLqZ*	*all2934*	2.08
Molybdopterin-guanine dinucleotide biosynthesis protein A	*mobA*	*ENSB:P-Yyl0n7CNfjZCd*	*all0961*	1.2
Diaminobutyrate-pyruvate transaminase	*dat*	*ENSB:9sHAdEXZrAhlq7B*	*all0396*	-1.14

Acetolactate synthase large subunit	*ilvB*	ENSB:MHH21VZ1XwX61yy	*all0427*	-1.25
Proton/sodium-glutamate symport protein	*dctA*	ENSB:TZqUO6daj0fFv6c	*all0342*	-1.08
Glycerate dehydrogenase	N/A	ENSB:zX_Ld1TTIPm3PX7	*all8087*	-2.56
**Photosynthesis and respiration**				
Homologue of the N-terminal domain of OCP	N/A	ENSB:Pjo4r6jSQqj9ZKS	*alr4783*	-1.22
Homolog of the C-terminal domain of the OCP	N/A	ENSB:-Skx2hMko_CWcds	*all4940*	-1.17
Bilin biosynthesis protein PecE	*pecE*	ENSB:QZBFZRurZXLurvC	*alr0526*	-1.52
Bilin biosynthesis protein PecF	*pecF*	ENSB:zGBsC3D8Gtj2ymD	*alr0527*	-1.58
Ferredoxin-1	*petF*	ENSB:cn6HVgj9et9zpgG	*all4148*	-1.49
Diflavin flavoprotein Flv4	*flv4*	ENSB:dd_Ejl4qMz5TVdO	*all4446*	-1.48
Pentapeptide repeat protein	N/A	ENSB:U7IgWWVcVHHMram	*alr5209*	-1.29
Two-component response regulator	N/A	ENSB:wQizH64rRL3JSms	*alr0072*	-1.01
**C-metabolism**				

Sbta bicarbonate Na+ symporter	*sbtA*	ENSB:Z3aL43jCkgjvSJl	*all2134*	-1.83
Sucrose synthase	*susB*	ENSB:49m5v0JcFmdYuMU	*all1059*	-1.16
Alpha.alpha-trehalase	*treH*	ENSB:lAnWugFkoPK758N	*all0166*	-1.43
Malto-oligosyltrehalose trehalohydrolase	*treZ*	ENSB:wYIPo5aOxCDd1v_	*all0168*	-1.65
Transketolase	*tkt_2*	ENSB:bvaFhPk4hqCTUJt	*all4052*	-1.51
Alpha-glucanotransferase	N/A	ENSB:8vPod86j-yWJPGD	*all0875*	-1.32
**Phosphor-metabolism**				
Phosphonate ABC transporter permease	N/A	ENSB:m4s0rAAffSgr4y7	*all8088*	-4.75
Phosphonate ABC transporter. phosphate-binding protein	N/A	ENSB:PbMDqwXP9BtwdTI	*all8089*	-5.41
Phosphonate ABC transporter. ATP-binding component	*phnL*	ENSB:LSu_SJZWdPPP-RV	*all2217*	-1.3
Phosphonate ABC transporter permease protein	*phnE*	ENSB:e6bTBbIW4RXbv2I	*all2227*	-1.32
ABC transporter. phosphate-binding protein; PhnD	*phnD*	ENSB:eQSxkgHwKX25u-K	*all2228*	-1.03
ABC tranporter. ATP-binding component	*phnC1*	ENSB:bURWmyKBm4Qc7CU	*all2230*	-1.9

Phosphodiesterase/alkaline phosphatase D	*phoD*	ENSB:hgDoJVzVX8lePKi	*alr2234*	-1.45
**Metals**				
Cation-efflux system membrane protein	N/A	ENSB:jOF_mylngWua0Ez	*all2845*	-1.37
Similar to Na+/H+ antiporter	N/A	ENSB:9_uVQJPEWe0wqUI	*all4832*	-1.22
ABC transporter. ATP-binding protein	*mntA*	ENSB:PmuYV0bqwlKOUDW	*all3575*	-1.07
**Transcription factors**				
PacR	*rbcR*	ENSB:3znthW3skDOmleL	*all3953*	-4.53

Table S5. Differentially expressed genes at t= 1 h, log_2FC (Δ*pacR*/CS). Genes marked in thick lines are directly bound by PacR according to Picossi et al. 2015.

**Category**	**ID**	**ENSEMBL_ID**	**Cyanobase_ID**	**log_2FC (ΔpacR/CS)**
**N-metabolism**				
Urease accessory protein	*ureG*	ENSB:thD7sRcdm3nPz9H	*alr0735*	2.43
Glutaminase 1	*glsA1*	ENSB:fjcIIBqee15rLqZ	*all2934*	1.54
Molybdopterin-guanine dinucleotide biosynthesis protein A	*mobA*	ENSB:P-Yyl0n7CNfjZCd	*all0961*	1.08
Diaminobutyrate-pyruvate transaminase	*dat*	ENSB:9sHAdEXZrAhlq7B	*all0396*	-3.29
Proton/sodium-glutamate symport protein	*dctA*	ENSB:TZqUO6daj0fFv6c	*all0342*	-2.24
Nitrite reductase	*nirA*	ENSB:e44ECOSAHPRWrlu	*alr0607*	-1.52
Nitrate transport nitrate-binding protein	*nrtA*	ENSB:V1D0skKAkHJgWSZ	*alr0608*	-1.44
Nitrate transport permease protein	*nrtB*	ENSB:7v-sc3mUDbuJK5G	*alr0609*	-1.62
Nitrate transport ATP-binding protein	*nrtC*	ENSB:Ys8OE0aTWeoXMXH	*alr0610*	-1.61
Nitrate transport ATP-binding protein	*nrtD*	ENSB:YD5wpg9hsgLEiVJ	*alr0611*	-1.62
Nitrate reductase	*narB*	ENSB:EYOY4XLgkxMnQT5	*alr0612*	-1.5

**Photosynthesis and respiration**				
NTD-OCP like protein	N/A	ENSB:YZqer7AeouGwj-a	*all4941*	-1.27
Photosystem II protein D1	*psbA3*	ENSB:9agEV78l86gFA_V	*alr4592*	-2.09
Photosystem II protein D1	*psbA1*	ENSB:yJKfo7_EpcR1xJq	*alr4866*	1.68
Light-independent protochlorophyllide reductase iron-sulfur ATP-binding protein	*chlL*	ENSB:O1DJvDa30jvN8hW	*all5078*	1.06
Ferredoxin-1	*petF*	ENSB:cn6HVgj9et9zpgG	*all4148*	-1.99
Diflavin flavoprotein Flv2	*flv2*	ENSB:txsyY6CJmI0pCJY	*all4444*	-1.29
Diflavin flavoprotein Flv4	*flv4*	ENSB:dd_Ejl4qMz5TVdO	*all4446*	-3.34
Pentapeptide repeat protein	N/A	ENSB:U7IgWWVcVHHMram	*alr5209*	-1.43
**C-metabolism**				
Sbta bicarbonate Na+ symporter	*sbtA*	ENSB:Z3aL43jCkgjvSJl	*all2134*	-4.33
Bicarbonate transport system permease protein	*cmpB*	ENSB:Q5MPpHiTaAnt52i	*alr2878*	-1.28
**Transcription Factors**				
PacR	*rbcR*	ENSB:3znthW3skDOmleL	*all3953*	-4.62

Cell wall-binding protein Fur	*furA*	ENSB:D3Qm5IOjvV5LGGN	*all1691*	1.14

Table S6. Differentially expressed genes at t= 12 h. log_2FC (Δ*pacR*/CS). Genes marked in thick lines are directly bound by PacR according to Picossi et al. 2015.

**Category**	**ID**	**ENSEMBL_ID**	**Cyanobase_ID**	**log_2FC (Δ*pacR*/CS)**
**N-metabolism**				
Urease accessory protein	*ureG*	*ENSB:thD7sRcdm3nPz9H*	*alr0735*	2.17
Glutaminase 1	*glsA1*	*ENSB:fjcIlBqee15rLqZ*	*all2934*	1.51
Molybdopterin-guanine dinucleotide biosynthesis protein A	*mobA*	*ENSB:P-Yyl0n7CNfjZCd*	*all0961*	1.11
Diaminobutyrate-pyruvate transaminase	*dat*	*ENSB:9sHAdEXZrAhlq7B*	*all0396*	-1.03
Alanine dehydrogenase	*ald*	*ENSB:rWYGwN8UWkGzo2q*	*alr2355*	1.24
Nitrogen stress-induced RNA 1	N/A	*ENSB:7BhRYdNgWHwPQX0*	N/A	2.08
Nitrogen stress-induced RNA 1	N/A	*ENSB:kLcNZRN7-pJLBxt*	N/A	1.7
Nitrogen stress-induced RNA 1	N/A	*ENSB:YhC5bG0zn0YQBkL*	N/A	1.63
**Heterocyst formation**				

Glycolipid synthase	*hglT*	*ENSB:5ThqEjI9sShuE-E*	*all5341*	3.27
Ketoacyl reductase	*hetN*	*ENSB:ZMQxbwAIyd9Q-fM*	*alr5358*	1.28
Glucosyltransferase	N/A	*ENSB:RmnPuja33XjbEKh*	*all2289*	1
Heterocyst envelope polysaccharide synthesis protein	*hepD*	*ENSB:Oc1V-X3m6qCq5eW*	*alr3698*	3.85
Glycosyltransferase	*hepE*	*ENSB:94sxNIFAyRkqwlV*	*alr3699*	1.43
Glucose-1-phosphate cytidylyltransferase	N/A	*ENSB:ZjfrPap_6rDqJvJ*	*alr2825*	4.91
Glycosyltransferase	N/A	*ENSB:aofJPhZKhvBy41C*	*alr2836*	3.39
Glycosyltransferase	N/A	*ENSB:ffiqyvkzMYyafmg*	*alr2839*	3.38
dTDP-4-dehydrorhamnose 3.5-epimerase	*rfbC*	*ENSB:CNlG9yMspfYs3EQ*	*alr2830*	4.09
Heterocyst differentiation ATP-binding protein	*hepA*	*ENSB:4TIFljsR-svhXZJ*	*alr2835*	3.49
Nitrogen fixation protein	*nifW*	*ENSB:KLm42LryWdoGB7J*	*all1433*	5.33
Nitrogenase molybdenum-iron protein beta chain	*nifN*	*ENSB:GgyBKW9hY4nEOAP*	*all1437*	3.74
Nitrogenase molybdenum-iron protein beta chain	*nifK*	*ENSB:p8L0nBpHJJRKNhu*	*all1440*	6.61
Nitrogenase molybdenum-iron protein alpha chain	*nifD*	*ENSB:SBhFQ-pgKfU865y*	*all1454*	4.78
Nitrogenase iron protein	*nifH1*	*ENSB:-UiwXBMw740um_c*	*all1455*	3.16

Nitrogen fixation protein	*nifU*	*ENSB:zD7Me4dVDd5AIk-*	*all1456*	2.9
Nitrogenase cofactor synthesis protein	*nifS*	*ENSB:cwZOUWtsHDDQELT*	*all1457*	3.64
Homocitrate synthase	*nifV1*	*ENSB:xnTLVRDJMQvACq9*	*alr1407*	3.65
Nitrogen fixation protein	*nifB*	*ENSB:oKdYkJwJmedayE7*	*all1517*	3.13
Protein HesB. heterocyst	*hesB*	*ENSB:0W1BDOVJav4vemq*	*all1431*	3.58
Protein HesA. heterocyst	*hesA*	*ENSB:Byut0LlNHsEQVLj*	*all1432*	2.65
Periplasmic [NiFeSe] hydrogenase large subunit	*hupL*	*ENSB:q7HshiWY1tEwz49*	*all0687*	3.5
Periplasmic [NiFe] hydrogenase small subunit	*hupS*	*ENSB:YybVoSB-3p1afrt*	*all0688*	2.93
Ferredoxin	*fdxB*	*ENSB:g-hu8fN-yJ9QOkL*	*asr2513*	2.56
Ferredoxin. heterocyst	*fdxH*	*ENSB:pgoFbyUO25Y11KZ*	*all1430*	3.18
Diflavin flavoprotein flv1B	*flv1B*	*ENSB:egKkM6AIKDL3lV3*	*all0177*	1.3
Putative diflavin flavoprotein flv3B	*flv3B*	*ENSB:gyAx2_YtlGWpP0r*	*all0178*	1.05
**C-metabolism**				
Sbta bicarbonate Na+ symporter	*sbtA*	*ENSB:Z3aL43jCkgjvSJl*	*all2134*	-1.08
6-Phosphofructokinase	*pfkA1*	*ENSB:STpcob1Kje9jW0R*	*all7335*	-1.11

Galactosyltransferase	N/A	*ENSB:F5rC4qxkMvFfiwq*	*all2037*	1.78
**Photosynthesis and respiration**				
Phycoerythrocyanin subunit beta	*pecB*	*ENSB:DlJg8tfpJb3Pc02*	*alr0523*	-1.98
Phycoerythrocyanin alpha chain	*pecA*	*ENSB:DkyiMvYmj7wtDsq*	*alr0524*	-1.85
Phycobilisome 34.5 kDa linker polypeptide. phycoerythrocyanin-associated. rod	*pecC*	*ENSB:4P_EwWN8WSSWyss*	*alr0525*	-2.06
Bilin biosynthesis protein PecE	*pecE*	*ENSB:QZBFZRurZXLurvC*	*alr0526*	-2.68
Bilin biosynthesis protein PecF	*pecF*	*ENSB:zGBsC3D8Gtj2ymD*	*alr0527*	-2.46
Photosystem II CP43 reaction center protein homologue	*isiA2*	*ENSB:n2bv19CnTlJ6ejC*	*all4002*	1.59
Photosystem II CP43 reaction center protein homologue	*isiA3*	*ENSB:72J4WLJgrkah1ix*	*all4003*	2.06
Oxygen-independent coproporphyrinogen III oxidase	*hemN*	*ENSB:Klj8zn66_ssKF-m*	*alr3126*	1.03
Ferredoxin-1	*petF*	*ENSB:cn6HVgj9et9zpgG*	*all4148*	-2.23
Flavodoxin I	*isiB*	*ENSB:vCA6KyCuxG-PG7-*	*alr2405*	1.94

Cytochrome c oxidase subunit 1	*coxA2*	*ENSB:6VxYhoGYWmvmjDB*	*alr2515*	3.22
Putative cytochrome c oxidase subunit 3	*coxC2*	*ENSB:Oyzg-naQZMZT0Ld*	*alr2516*	3.67
Cytochrome c oxidase subunit 1	*coxA3*	*ENSB:OjIeYMGIKj96usn*	*alr2732*	2.44
Oxidoreductase	N/A	*ENSB:_N-AKaQFkqo5q49*	*all5345*	3.28
Pentapeptide repeat protein	N/A	*ENSB:U7IgWWVcVHHMram*	*alr5209*	-1.43
**Phosphor-metabolism**				
Phosphonate ABC transporter. ATP-binding component	*phnK*	*ENSB:L4cUHY-SreLDCy2*	*all2218*	-1.01
ABC transporter. periplasmic phosphate-binding protein	N/A	*ENSB:ANyliMgnrcBMIfb*	*alr1094*	2.18
**Transcription factors**				
PacR	*rbcR*	*ENSB:3znthW3skDOmleL*	*all3953*	-4.85
Nitrogen-responsive regulatory protein	*ntcA*	*ENSB:wHiGICXo9Og7Xlz*	*alr4392*	1.12
RNA polymerase sigma-subunit	*sigC*	*ENSB:WGvPMBCl6Hl3Gwg*	*all1692*	1.1
AraC type Transcriptional regulator	N/A	*ENSB:f_q6Xin8pCUNvPv*	*all2035*	1.03

Table S7. Differentially expressed genes in the CS. log_2FC (t1/t0) and log_2FC (t12/t0). Genes marked in thick lines are directly bound by PacR according to Picossi et al. 2015.

**Category**	**ID**	**ENSEMBL_ID**	**Cyanobase_ID**	**log_2FC (t1/t0)**	**log_2FC (t12/t0)**
**N-metabolism**					
Leucine dehydrogenase	*ldh*	*ENSB:DCJLnD1-vRm8aTd*	*all0426*	-1.01	-2.45
Cyanophycinase	*cphB_1*	*ENSB:qnAFH-zZi1_Cg7h*	*all0571*	-4.01	-4.51
Urease accessory protein	*ureG*	*ENSB:thD7sRcdm3nPz9H*	*alr0735*	N/A	N/A
Glutaminase 1	*glsA1*	*ENSB:fjcIlBqee15rLqZ*	*all2934*	N/A	N/A
Molybdopterin-guanine dinucleotide biosynthesis protein A	*mobA*	*ENSB:P-Yyl0n7CNfjZCd*	*all0961*	N/A	N/A
Diaminobutyrate-pyruvate transaminase	*dat*	*ENSB:9sHAdEXZrAhlq7B*	*all0396*	N/A	N/A
Acetolactate synthase large subunit	*ilvB*	*ENSB:MHH21VZ1XwX61yy*	*all0427*	-1.08	-1.86
Proton/sodium-glutamate symport protein	*dctA*	*ENSB:TZqUO6daj0fFv6c*	*all0342*	N/A	N/A
Glycerate dehydrogenase	N/A	*ENSB:zX_Ld1TTIPm3PX7*	*all8087*	-3.45	-2.36
Alanine dehydrogenase	*ald*	*ENSB:rWYGwN8UWkGzo2q*	*alr2355*	N/A	N/A

Glutamine synthetase	*glnA*	*ENSB:ab0fhVsYEpHjH88*	*alr2328*	N/A	2.11
Nitrate transport nitrate-binding protein	*nrtA*	*ENSB:V1D0skKAkHJgWSZ*	*alr0608*	4.58	5.37
Nitrate transport permease protein	*nrtB*	*ENSB:7v-sc3mUDbuJK5G*	*alr0609*	4.55	5.15
Nitrate transport ATP-binding protein	*nrtC*	*ENSB:Ys8OE0aTWeoXMXH*	*alr0610*	4.49	4.94
Nitrate transport ATP-binding protein	*nrtD*	*ENSB:YD5wpg9hsgLEiVJ*	*alr0611*	4.45	4.88
Nitrate reductase	*narB*	*ENSB:EYOY4XLgkxMnQT5*	*alr0612*	3.04	3.44
Nitrite reductase	*nirA*	*ENSB:e44ECOSAHPRWrlu*	*alr0607*	4.59	5.43
Nitrogen stress-induced RNA 1	N/A	*ENSB:7BhRYdNgWHwPQX0*	N/A	N/A	1.74
Nitrogen stress-induced RNA 1	N/A	*ENSB:kLcNZRN7-pJLBxt*	N/A	N/A	N/A
Nitrogen stress-induced RNA 1	N/A	*ENSB:YhC5bG0zn0YQBkL*	N/A	N/A	1.04
**Heterocyst formation**					
Glycolipid synthase	*hglT*	*ENSB:5ThqEjI9sShuE-E*	*all5341*	N/A	N/A
Ketoacyl reductase	*hetN*	*ENSB:ZMQxbwAIyd9Q-fM*	*alr5358*	N/A	N/A
Glucosyltransferase	N/A	*ENSB:RmnPuja33XjbEKh*	*all2289*	N/A	1.96

Heterocyst envelope polysaccharide synthesis protein	*hepD*	*ENSB:Oc1V-X3m6qCq5eW*	*alr3698*	N/A	N/A
Glycosyltransferase	*hepE*	*ENSB:94sxNIFAyRkqwlV*	*alr3699*	N/A	N/A
Glucose-1-phosphate cytidylyltransferase	N/A	*ENSB:ZjfrPap_6rDqJvJ*	*alr2825*	N/A	1.3
Glycosyltransferase	N/A	*ENSB:aofJPhZKhvBy41C*	*alr2836*	N/A	N/A
Glycosyltransferase	N/A	*ENSB:ffiqyvkzMYyafmg*	*alr2839*	N/A	1.03
dTDP-4-dehydrorhamnose 3.5-epimerase	*rfbC*	*ENSB:CNlG9yMspfYs3EQ*	*alr2830*	N/A	1.07
Heterocyst differentiation ATP-binding protein	*hepA*	*ENSB:4TIFljsR-svhXZJ*	*alr2835*	N/A	N/A
Nitrogen fixation protein	*nifW*	*ENSB:KLm42LryWdoGB7J*	*all1433*	N/A	N/A
Nitrogenase molybdenum iron protein beta chain	*nifN*	*ENSB:GgyBKW9hY4nEOAP*	*all1437*	N/A	N/A
Nitrogenase molybdenum iron protein beta chain	*nifK*	*ENSB:p8L0nBpHJJRKNhu*	*all1440*	N/A	N/A
Nitrogenase molybdenum iron protein alpha chain	*nifD*	*ENSB:SBhFQ-pgKfU865y*	*all1454*	N/A	N/A
Nitrogenase iron protein	*nifH1*	*ENSB:-UiwXBMw740um_c*	*all1455*	N/A	N/A

Nitrogen fixation protein	*nifU*	*ENSB:zD7Me4dVDd5AIk-*	*all1456*	N/A	N/A
Nitrogenase cofactor synthesis protein	*nifS*	*ENSB:cwZOUWtsHDDQELT*	*all1457*	N/A	N/A
Homocitrate synthase	*nifV1*	*ENSB:xnTLVRDJMQvACq9*	*alr1407*	N/A	N/A
Nitrogen fixation protein	*nifB*	*ENSB:oKdYkJwJmedayE7*	*all1517*	N/A	N/A
Protein HesB. heterocyst	*hesB*	*ENSB:0W1BDOVJav4vemq*	*all1431*	N/A	N/A
Protein HesA. heterocyst	*hesA*	*ENSB:Byut0LlNHsEQVLj*	*all1432*	1.83	2.92
Periplasmic [NiFeSe] hydrogenase large subunit	*hupL*	*ENSB:q7HshiWY1tEwz49*	*all0687*	N/A	N/A
Periplasmic [NiFe] hydrogenase small subunit	*hupS*	*ENSB:YybVoSB-3p1afrt*	*all0688*	N/A	N/A
Ferredoxin	*fdxB*	*ENSB:g-hu8fN-yJ9QOkL*	*asr2513*	N/A	N/A
Ferredoxin. heterocyst	*fdxH*	*ENSB:pgoFbyUO25Y11KZ*	*all1430*	N/A	N/A
Diflavin flavoprotein flv1B	*flv1B*	*ENSB:egKkM6AIKDL3lV3*	*all0177*	N/A	N/A
Diflavin flavoprotein flv3B	*flv3B*	*ENSB:gyAx2_YtlGWpP0r*	*all0178*	N/A	N/A
**Photosynthesis and respiration**					

NTD-OCP like protein	N/A	*ENSB:YZqer7AeouGwj-a*	*all4941*	1.89	-1.62
Homologue of the N-terminal domain of OCP	N/A	*ENSB:Pjo4r6jSQqj9ZKS*	*alr4783*	-3.58	-3.53
Homolog of the C-terminal domain of the OCP	N/A	*ENSB:-Skx2hMko_CWcds*	*All4940*	-2.18	-2.11
Phycoerythrocyanin subunit beta	*pecB*	*ENSB:DlJg8tfpJb3Pc02*	*alr0523*	-3.9	N/A
Phycoerythrocyanin alpha chain	*pecA*	*ENSB:DkyiMvYmj7wtDsq*	*alr0524*	-3.97	N/A
Phycobilisome 34.5 kDa linker polypeptide. phycoerythrocyanin-associated. rod	*pecC*	*ENSB:4P_EwWN8WSSWyss*	*alr0525*	-4.41	N/A
Bilin biosynthesis protein	*pecE*	*ENSB:QZBFZRurZXLurvC*	*alr0526*	-4.65	N/A
Bilin biosynthesis protein	*pecF*	*ENSB:zGBsC3D8Gtj2ymD*	*alr0527*	-3.69	N/A
Photosystem II protein D1	*psbA3*	*ENSB:9agEV78l86gFA_V*	*alr4592*	2.61	-1.82
Photosystem II protein D1	*psbA1*	*ENSB:yJKfo7_EpcR1xJq*	*alr4866*	N/A	N/A
Photosystem II CP43 reaction center protein homologue	*isiA2*	*ENSB:n2bv19CnTlJ6ejC*	*all4002*	N/A	N/A
Photosystem II CP43 reaction center protein homologue	*isiA3*	*ENSB:72J4WLJgrkah1ix*	*all4003*	N/A	N/A

Light-independent protochlorophyllide reductase iron-sulfur ATP-binding protein	*chlL*	*ENSB:O1DJvDa30jvN8hW*	*all5078*	-2.65	N/A
Oxygen-independent coproporphyrinogen III oxidase	*hemN*	*ENSB:Klj8zn66_ssKF-m*	*alr3126*	N/A	N/A
Ferredoxin-1	*petF*	*ENSB:cn6HVgj9et9zpgG*	*all4148*	N/A	N/A
Flavodoxin	*isiB*	*ENSB:vCA6KyCuxG-PG7-*	*alr2405*	N/A	N/A
Diflavin flavoprotein flv2	*flv2*	*ENSB:txsyY6CJmI0pCJY*	*all4444*	1.01	N/A
Diflavin flavoprotein Flv4	*flv4*	*ENSB:dd_Ejl4qMz5TVdO*	*all4446*	N/A	-1.14
Cytochrome c oxidase subunit 1	*CoxA2*	*ENSB:6VxYhoGYWmvmjDB*	*alr2515*	N/A	N/A
Putative cytochrome c oxidase subunit 3	*coxC2*	*ENSB:Oyzg-naQZMZT0Ld*	*alr2516*	N/A	N/A
Cytochrome c oxidase subunit 1	*coxA3*	*ENSB:OjIeYMGIKj96usn*	*alr2732*	N/A	N/A
Oxidoreductase	N/A	*ENSB:_N-AKaQFkqo5q49*	*all5345*	N/A	N/A
Pentapeptide repeat protein	N/A	*ENSB:U7IgWWVcVHHMram*	*alr5209*	N/A	N/A
Two-component response regulator	N/A	*ENSB:wQizH64rRL3JSms*	*alr0072*	-2.1	-1.99
**C-metabolism**					

Sbta bicarbonate Na+ symporter	*sbtA*	*ENSB:Z3aL43jCkgjvSJI*	*all2134*	2.27	N/A
Bicarbonate transport system permease protein	*cmpB*	*ENSB:Q5MPpHiTaAnt52i*	*alr2878*	1.19	N/A
Sucrose synthase	*susB*	*ENSB:49m5v0JcFmdYuMU*	*all1059*	-4.13	-4.2
Alpha.alpha- trehalase	*treH*	*ENSB:lAnWugFkoPK758N*	*all0166*	-3.55	-4.02
Malto-oligosyltrehalose trehalohydrolase	*treZ*	*ENSB:wYIPo5aOxCDd1v_*	*all0168*	-4.28	-5.15
Transketolase	*tkt_2*	*ENSB:bvaFhPk4hqCTUJt*	*all4052*	-4.3	-4.68
Alpha-glucanotransferase	N/A	*ENSB:8vPod86j-yWJPGD*	*all0875*	-5.6	-6.42
6-Phosphofructokinase	*pfkA1*	*ENSB:STpcob1Kje9jW0R*	*all7335*	N/A	1.23
Galactosyltransferase	N/A	*ENSB:F5rC4qxkMvFfiwq*	*all2037*	N/A	N/A
**Metals**					
Cation-efflux system membrane protein	N/A	*ENSB:jOF_mylngWua0Ez*	*all2845*	-2.02	-2.47
Similar to Na+/H+ antiporter	N/A	*ENSB:9_uVQJPEWe0wqUI*	*all4832*	-2.59	-3.01
ABC transporter. ATP-binding protein	*mntA*	*ENSB:PmuYV0bqwlKOUDW*	*all3575*	N/A	-1.78

**Phosphor-metabolism**					
Phosphonate ABC transporter permease	N/A	*ENSB:m4s0rAAffSgr4y7*	*all8088*	-4.21	-4.33
Phosphonate ABC transporter. phosphate-binding protein	N/A	*ENSB:PbMDqwXP9BtwdTI*	*all8089*	-5.68	-1.88
ATP-binding component	*phnL*	*ENSB:LSu_SJZWdPPP-RV*	*all2217*	-1.32	-1.34
phosphonate ABC transporter. ATP-binding component	*phnK*	*ENSB:L4cUHY-SreLDCy2*	*all2218*	N/A	N/A
phosphonate ABC transporter permease protein	*phnE*	*ENSB:e6bTBbIW4RXbv2I*	*all2227*	N/A	N/A
ABC tranporter. phosphate-binding protein; PhnD	*phnD*	*ENSB:eQSxkgHwKX25u-K*	*all2228*	-1.56	-1.88
ABC transporter. ATP-binding component	*phnC1*	*ENSB:bURWmyKBm4Qc7CU*	*all2230*	-3.93	-3.4
Phosphodiesterase/alkaline phosphatase D	*phoD*	*ENSB:hgDoJVzVX8lePKi*	*alr2234*	-2	-2.89
ABC transporter. periplasmic phosphate-binding protein	N/A	*ENSB:ANyliMgnrcBMIfb*	*alr1094*	N/A	N/A
**Transcription Factors**					
PacR	*rbcR*	*ENSB:3znthW3skDOmleL*	*all3953*	N/A	N/A
Nitrogen-responsive regulatory protein	*ntcA*	*ENSB:wHiGICXo9Og7Xlz*	*alr4392*	N/A	N/A

Cell wall-binding protein Fur	*furA*	*ENSB:D3Qm5IOjvV5LGGN*	*all1691*	-1.3	N/A
RNA polymerase sigma-subunit	*sigC*	*ENSB:WGvPMBCl6Hl3Gwg*	*all1692*	N/A	1.8
AraC type Transcriptional regulator	N/A	*ENSB:f_q6Xin8pCUNvPv*	*all2035*	N/A	3.13

Table S8. Differentially expressed genes found in the Δ*pacR* mutant. log_2FC (t1/t0) and log_2FC (t12/t0). Genes marked in thick lines are directly bound by PacR according to Picossi et al. 2015.

**Category**	**ID**	**ENSEMBL_ID**	**Cyanobase_ID**	**log_2FC (t1/t0)**	**log_2FC (t12/t0)**
**N-metabolism**					
Leucine dehydrogenase	*ldh*	*ENSB:DCJLnD1-vRm8aTd*	*all0426*	N/A	N/A
Cyanophycinase	*cphB_1*	*ENSB:qnAFH-zZi1_Cg7h*	*all0571*	-2.36	-2.96
Urease accessory protein	*ureG*	*ENSB:thD7sRcdm3nPz9H*	*alr0735*	N/A	N/A
Glutaminase 1	*glsA1*	*ENSB:fjcIlBqee15rLqZ*	*all2934*	N/A	N/A
Molybdopterin-guanine dinucleotide biosynthesis protein A	*mobA*	*ENSB:P-Yyl0n7CNfjZCd*	*all0961*	N/A	N/A

Diaminobutyrate-pyruvate transaminase	*dat*	*ENSB:9sHAdEXZrAhlq7B*	*all0396*	N/A	N/A
Acetolactate synthase large subunit	*ilvB*	*ENSB:MHH21VZ1XwX61yy*	*all0427*	N/A	N/A
Proton/sodium-glutamate symport protein	*dctA*	*ENSB:TZqUO6daj0fFv6c*	*all0342*	N/A	N/A
Glycerate dehydrogenase	N/A	*ENSB:zX_Ld1TTIPm3PX7*	*all8087*	N/A	N/A
Alanine dehydrogenase	*ald*	*ENSB:rWYGwN8UWkGzo2q*	*alr2355*	N/A	1.52
Glutamine synthetase	*glnA*	*ENSB:ab0fhVsYEpHjH88*	*alr2328*	1	1.96
Nitrate transport nitrate-binding protein	*nrtA*	*ENSB:V1D0skKAkHJgWSZ*	*alr0608*	4.08	6.26
Nitrate transport permease protein	*nrtB*	*ENSB:7v-sc3mUDbuJK5G*	*alr0609*	3.84	5.98
Nitrate transport ATP-binding protein	*nrtC*	*ENSB:Ys8OE0aTWeoXMXH*	*alr0610*	3.97	5.93
Nitrate transport ATP-binding protein	*nrtD*	*ENSB:YD5wpg9hsgLEiVJ*	*alr0611*	4.06	6.01
Nitrate reductase	*narB*	*ENSB:EYOY4XLgkxMnQT5*	*alr0612*	2.09	3.92
Nitrite reductase	*nirA*	*ENSB:e44ECOSAHPRWrlu*	*alr0607*	4.07	6.49
Nitrogen stress-induced RNA 1	N/A	*ENSB:7BhRYdNgWHwPQX0*	N/A	N/A	3.97
Nitrogen stress-induced RNA 1	N/A	*ENSB:kLcNZRN7-pJLBxt*	N/A	N/A	1.86

Nitrogen stress-induced RNA 1	N/A	*ENSB:YhC5bG0zn0YQBkL*	N/A	N/A	2.63
**Heterocyst formation**					
Glycolipid synthase	*hglT*	*ENSB:5ThqEjI9sShuE-E*	*all5341*	N/A	2.98
Ketoacyl reductase	*hetN*	*ENSB:ZMQxbwAIyd9Q-fM*	*alr5358*	N/A	N/A
Glucosyltransferase	N/A	*ENSB:RmnPuja33XjbEKh*	*all2289*	N/A	2.41
Heterocyst envelope polysaccharide synthesis protein	*hepD*	*ENSB:Oc1V-X3m6qCq5eW*	*alr3698*	N/A	3.89
Glycosyltransferase	*hepE*	*ENSB:94sxNIFAyRkqwIV*	*alr3699*	N/A	1.38
Glucose-1-phosphate cytidylyltransferase	N/A	*ENSB:ZjfrPap_6rDqJvJ*	*alr2825*	N/A	6.95
Glycosyltransferase	N/A	*ENSB:aofJPhZKhvBy41C*	*alr2836*	N/A	3.66
Glycosyltransferase	N/A	*ENSB:ffiqyvkzMYyafmg*	*alr2839*	N/A	4.3
dTDP-4-dehydrorhamnose 3.5-epimerase	*rfbC*	*ENSB:CNlG9yMspfYs3EQ*	*alr2830*	N/A	5.09
Heterocyst differentiation ATP-binding protein HepA	*hepA*	*ENSB:4TIFljsR-svhXZJ*	*alr2835*	N/A	4.08
Nitrogen fixation protein	*nifW*	*ENSB:KLm42LryWdoGB7J*	*all1433*	N/A	3.07

Nitrogenase molybdenum-iron protein beta chain	*nifN*	*ENSB:GgyBKW9hY4nEOAP*	*all1437*	N/A	3.87
Nitrogenase molybdenum-iron protein beta chain	*nifK*	*ENSB:p8L0nBpHJJRKNhu*	*all1440*	N/A	5.65
Nitrogenase molybdenum-iron protein alpha chain	*nifD*	*ENSB:SBhFQ-pgKfU865y*	*all1454*	N/A	4.13
Nitrogenase iron protein 1	*nifH1*	*ENSB:-UiwXBMw740um_c*	*all1455*	N/A	3.19
Nitrogen fixation protein	*nifU*	*ENSB:zD7Me4dVDd5AIk-*	*all1456*	N/A	2.11
Nitrogenase cofactor synthesis protein NifS	*nifS*	*ENSB:cwZOUWtsHDDQELT*	*all1457*	N/A	2.49
Homocitrate synthase	*nifV1*	*ENSB:xnTLVRDJMQvACq9*	*alr1407*	N/A	4.53
Nitrogen fixation protein	*nifB*	*ENSB:oKdYkJwJmedayE7*	*all1517*	N/A	2.95
Protein HesB. heterocyst	*hesB*	*ENSB:0W1BDOVJav4vemq*	*all1431*	N/A	4.23
Protein HesA. heterocyst	*hesA*	*ENSB:Byut0LINHsEQVLj*	*all1432*	1.79	5.27
Periplasmic [NiFeSe] hydrogenase large subunit	*hupL*	*ENSB:q7HshiWY1tEwz49*	*all0687*	N/A	3.49
Periplasmic [NiFe] hydrogenase small subunit	*hupS*	*ENSB:YybVoSB-3p1afrt*	*all0688*	N/A	2.23

Ferredoxin	*fdxB*	*ENSB:g-hu8fN-yJ9QOkL*	*asr2513*	N/A	2.91
Ferredoxin. heterocyst	*fdxH*	*ENSB:pgoFbyUO25Y11KZ*	*all1430*	N/A	3.25
Diflavin flavoprotein flv1B	*flv1B*	*ENSB:egKkM6AIKDL3lV3*	*all0177*	N/A	1.02
Diflavin flavoprotein flv3B	*flv3B*	*ENSB:gyAx2_YtlGWpP0r*	*all0178*	N/A	1.32
**Photosynthesis and respiration**					
NTD-OCP like protein	N/A	*ENSB:YZqer7AeouGwj-a*	*all4941*	1.38	N/A
Homologue of the N-terminal domain of OCP	N/A	*ENSB:Pjo4r6jSQqj9ZKS*	*alr4783*	-1.77	-2
Homolog of the C-terminal domain of the OCP	N/A	*ENSB:-Skx2hMko_CWcds*	*all4940*	-1.03	N/A
Phycoerythrocyanin subunit beta	*pecB*	*ENSB:DlJg8tfpJb3Pc02*	*alr0523*	-2.57	-1.92
Phycoerythrocyanin alpha chain	*pecA*	*ENSB:DkyiMvYmj7wtDsq*	*alr0524*	-2.42	-1.89
Phycobilisome 34.5 kDa linker polypeptide. phycoerythrocyanin-associated. rod	*pecC*	*ENSB:4P_EwWN8WSSWyss*	*alr0525*	-2.75	-2.03
Bilin biosynthesis protein PecE	*pecE*	*ENSB:QZBFZRurZXLurvC*	*alr0526*	-3.16	-2.31

Bilin biosynthesis protein PecF	*pecF*	*ENSB:zGBsC3D8Gtj2ymD*	*alr0527*	-1.94	-2.2
Photosystem II protein D1	*psbA3*	*ENSB:9agEV78l86gFA_V*	*alr4592*	1.06	-1.28
Photosystem II protein D1	*psbA1*	*ENSB:yJKfo7_EpcR1xJq*	*alr4866*	N/A	N/A
Photosystem II CP43 reaction center protein homologue	*isiA2*	*ENSB:n2bv19CnTlJ6ejC*	*all4002*	N/A	1.89
Photosystem II CP43 reaction center protein homologue	*isiA3*	*ENSB:72J4WLJgrkah1ix*	*all4003*	N/A	1.76
Light-independent protochlorophyllide reductase iron-sulfur ATP-binding protein	*chlL*	*ENSB:O1DJvDa30jvN8hW*	*all5078*	-1.54	N/A
Oxygen-independent coproporphyrinogen III oxidase	*hemN*	*ENSB:Klj8zn66_ssKF-m*	*alr3126*	N/A	N/A
Ferredoxin-1	*petF*	*ENSB:cn6HVgj9et9zpgG*	*all4148*	N/A	N/A
Flavodoxin	*isiB*	*ENSB:vCA6KyCuxG-PG7-*	*alr2405*	N/A	1.8
Diflavin flavoprotein Flv2	*flv2*	*ENSB:txsyY6CJmI0pCJY*	*all4444*	N/A	N/A
Diflavin flavoprotein Flv4	*flv4*	*ENSB:dd_Ejl4qMz5TVdO*	*all4446*	N/A	N/A
Cytochrome c oxidase subunit 1	*coxA2*	*ENSB:6VxYhoGYWmvmjDB*	*alr2515*	N/A	3.14

Putative cytochrome c oxidase subunit 3	*coxC2*	*ENSB:Oyzg-naQZMZT0Ld*	*alr2516*	N/A	4.16
Cytochrome c oxidase subunit 1	*coxA3*	*ENSB:OjIeYMGIKj96usn*	*alr2732*	N/A	3.25
Oxidoreductase	N/A	*ENSB:_N-AKaQFkqo5q49*	*all5345*	N/A	3.06
Pentapeptide repeat protein	N/A	*ENSB:U7IgWWVcVHHMram*	*alr5209*	N/A	N/A
Two-component response regulator	N/A	*ENSB:wQizH64rRL3JSms*	*alr0072*	-2.1	-1.99
**C-metabolism**					
Sbta bicarbonate Na+ symporter	*sbtA*	*ENSB:Z3aL43jCkgjvSJI*	*all2134*	N/A	N/A
Bicarbonate transport system permease protein	*cmpB*	*ENSB:Q5MPpHiTaAnt52i*	*alr2878*	N/A	N/A
Sucrose synthase	*susB*	*ENSB:49m5v0JcFmdYuMU*	*all1059*	-2.18	-2.06
Alpha.alpha-trehalase	*treH*	*ENSB:lAnWugFkoPK758N*	*all0166*	-1.79	-1.94
Malto-oligosyltrehalose trehalohydrolase	*treZ*	*ENSB:wYIPo5aOxCDd1v_*	*all0168*	-2.27	-1.98
Transketolase	*tkt_2*	*ENSB:bvaFhPk4hqCTUJt*	*all4052*	-2.4	-2.21
Alpha-glucanotransferase	N/A	*ENSB:8vPod86j-yWJPGD*	*all0875*	-3.46	-3.96
6-Phosphofructokinase	*pfkA1*	*ENSB:STpcob1Kje9jW0R*	*all7335*	N/A	N/A

Galactosyltransferase	N/A	*ENSB:F5rC4qxkMvFfiwq*	*all2037*	N/A	1.91
**Metals**					
Cation-efflux system membrane protein	N/A	*ENSB:jOF_mylngWua0Ez*	*all2845*	N/A	N/A
Similar to Na+/H+ antiporter	N/A	*ENSB:9_uVQJPEWe0wqUI*	*all4832*	N/A	-1.27
ABC transporter. ATP-binding protein	*mntA*	*ENSB:PmuYV0bqwlKOUDW*	*all3575*	N/A	-1.13
**Phosphor-metabolism**					
Phosphonate ABC transporter permease	N/A	*ENSB:m4s0rAAffSgr4y7*	*all8088*	N/A	N/A
Phosphonate ABC transporter. phosphate-binding protein	N/A	*ENSB:PbMDqwXP9BtwdTI*	*all8089*	N/A	N/A
ATP-binding component	*phnL*	*ENSB:LSu_SJZWdPPP-RV*	*all2217*	N/A	N/A
Phosphonate ABC transporter. ATP-binding component	*phnK*	*ENSB:L4cUHY-SreLDCy2*	*all2218*	N/A	N/A
Phosphonate ABC transporter permease protein	*phnE*	*ENSB:e6bTBbIW4RXbv2I*	*all2227*	N/A	N/A
ABC tranporter. phosphate-binding protein	*phnD*	*ENSB:eQSxkgHwKX25u-K*	*all2228*	N/A	N/A

ABC tranporter. ATP-binding component	*phnC1*	*ENSB:bURWmyKBm4Qc7CU*	*all2230*	N/A	N/A
Phosphodiesterase. alkaline phosphatase D	*phoD*	*ENSB:hgDoJVzVX8lePKi*	*alr2234*	N/A	N/A
ABC transporter. periplasmic phosphate-binding protein	N/A	*ENSB:ANyliMgnrcBMIfb*	*alr1094*	N/A	2.58
**Transcription Factors**					
PacR	*rbcR*	*ENSB:3znthW3skDOmleL*	*all3953*	N/A	N/A
Nitrogen-responsive regulatory protein	*ntcA*	*ENSB:wHiGICXo9Og7Xlz*	*alr4392*	N/A	N/A
Cell wall-binding protein Fur	*furA*	*ENSB:D3Qm5IOjvV5LGGN*	*all1691*	N/A	N/A
RNA polymerase sigma-subunit	*sigC*	*ENSB:WGvPMBCl6Hl3Gwg*	*all1692*	N/A	2.56
AraC type transcriptional regulator	N/A	*ENSB:f_q6Xin8pCUNvPv*	*all2035*	N/A	3.02
